# Mathematical modelling of inflammatory process and obesity in osteoarthritis

**DOI:** 10.1371/journal.pone.0323258

**Published:** 2025-06-02

**Authors:** Juntong Lai, Damien Lacroix

**Affiliations:** 1 Insigneo Institute, University of Sheffield, Sheffield, United Kingdom; 2 School of Mechanical, Aerospace and Civil Engineering, University of Sheffield, Sheffield, United Kingdom; Texas A&M University-San Antonio, UNITED STATES OF AMERICA

## Abstract

Osteoarthritis (OA) is prevalent in obese people due to the inflamed adipose tissue surrounding the joints. The increase in obesity level upregulates adipokines enhancing inflammation. Whilst a few main inflammatory mediators including cytokines and adipokines have been identified, the multi-effects of obesity and exercise on OA inflammation are elusive. This study aimed to develop a five-variable mathematical model elucidating the dynamics of OA inflammation associated with obesity and physical activity. Within this model, pro- and anti-inflammatory cytokines, adipokines, matrix metalloproteinases and fibronectin fragments interact to regulate the inflammatory process. The damage of cartilage is considered crucial to stimulate the production of fibronectin fragments, subsequently leading to chronic inflammation. The adipokine production is dependent on the obesity level measured by body mass index (BMI). Hill functions are used to describe the interactions (stimulation and inhibition) between mediators and the nonlinear impacts of physical activity level on adiposity. The dynamics of this inflammation system was verified and analysed through bifurcation diagrams. Results indicate that a high BMI reduces the bistability of the system up to a BMI value of 33 for which inflammation is persistent in the non-dimensionalised model. In codimension-2 bifurcations, parameters of adipokine production can govern the transition of system behaviours. This shows the variability of individuals susceptible to OA inflammation related to obesity. The minimum damage leading to persistent inflammation is decreased as BMI increases and the correlation is nonlinear, which suggests a significant rise in OA risk with a high level of obesity. Additionally, the simulations of multiple physical activity intervention strategies suggest that physical activity can minimise and postpone inflammation by downregulating adipokines within a window period after injury. This novel computational model describes the roles of obesity and physical activity in OA inflammation, providing a mathematical framework to evaluate the risk of OA inflammation from the perspective of obesity.

## Introduction

As a multi-factorial disease of the entire joint, osteoarthritis (OA) progressively causes the loss of joint function and pain along with tissue degeneration due to the unbalance of metabolic chronic inflammation and abnormal biomechanics. Concomitantly, disability and comorbidities occur with OA development [1], imposing significant medical burden worldwide [2,3]. The number of patients with OA worldwide will reach more than 600 million by 2050 [4]. Obesity is a predominant risk factor aggravating the inflammation and load within the joint [5–7]. The rapidly increasing prevalence of obesity [8] will lead to severe challenges on remitting medical burden from OA. Obesity significantly rises the joint force and undermines the biomechanics of the joints [9–12], whereas the awareness of the regulatory mechanism of obesity-associated OA inflammation is still limited. Regardless of that, the best strategies to manage the disease are still prevention and intervention of early OA such as controlling body weight and physical activity [13–15]. Compared to obesity commonly measured by body mass index (BMI), adiposity refers to the amount of body fat that plays a pivotal role in the inflammatory process [16]. Physical activity level (PAL) and nutritional control can reduce adiposity and increase muscle strength, resulting in weight loss for obese individuals [17]. Physiological loading is sensitive to body weight within the load-bearing joints [18]. However, conclusive evidence linking physical activity to the reduction of body weight is absent [19,20]. This suggests that exercise might not have a direct benefit on the joint by reducing the inherent mechanical loading. Instead, physical activity might exert a more significant impact on modulating the serum level of inflammatory mediators [21,22] in the context of OA. The variation of adiposity due to physical activity might be responsible for this impact on OA inflammation.

Low-grade inflammation is present at early stage of OA in prior to structural changes [23–25], though no evidence shows that inflammation predisposes to the onset of OA as various risk factors, such as age, gender, genetics, obesity, nutrition and mechanics, might concurrently trigger OA. In general, the presence of inflammation can recruit specialised cells to repair the rupture tissue of cartilage. The lack of vascular system nevertheless results in the unbalance of inflammatory metabolism [26–29], hence cartilage tissue is eventually degraded along with chronic inflammation where multiple soluble mediators interact, including pro- and anti-inflammatory cytokines (PICs and AICs) [30], matrix metalloproteinases (MMPs) [31–33] and adipokines [34–37]. The potent production of PICs can be activated by the tissue breakdown including fibronectin-fragments (Fn-fs) [38]. As the PIC level increases, MMPs are secreted to catabolise the extracellular matrix (ECM) of the cartilage and AICs are released to reduce the active inflammatory activities. Furthermore, adipose tissue is dispersed across the whole body and it can systemically and locally contribute to the OA metabolism and joint biomechanics [16,39]. Specifically, adipokines primarily derived from adipose tissue are an important class of soluble mediators associated with OA [39]. Adipokines can stimulate the production of inflammatory mediators such as PICs and MMPs [35,37,40–42]. The accumulation of adipose tissue leads to an excess of adipokines, which can be responsible for the metabolic effects of obesity on OA progression.

Computational approaches have demonstrated the potential to mimic the cartilage degradation and inflammatory process [43–52]. However, both mechanistic and data-driven modelling approaches are still limited by the complex molecular and cellular crosstalk. This makes it challenging to explain the precise mechanism of OA. A few attempts [53–55] are emerging to integrate knowledge-based and data-driven modelling approaches to explain the network of signal transduction of OA. The computation of signal transduction heavily relies on available concluded biological data, thereby underscoring the necessity for incorporating current knowledge of OA [56]. However, parameter complexity escalates with the increased number of signalling pathways [57]. As an alternative to study the role of cytokines in OA inflammation and to reduce the computing complexity, Baker *et al*. extended a two-variable RA model of cytokines [58] to a general four-variable OA model of cartilage inflammation [43]. Distinct to the autoimmune disease, rheumatoid arthritis (RA) characterised by severe synovial inflammation, OA involves low-grade inflammation due to adaptive and innate immune responses under mechanical stimuli [59]. Although inflammation is found in both diseases, the molecular inflammatory mechanisms are different. This extension provided a representative model with relatively low computational complexity to analyse molecular interplays in particular for those biological regulations lacking sufficient data support. Four characteristics of the inflammation system (homeostasis, persistent inflammation, bistable and tristable inflammation) were found and the uncertainty of estimated parameters to alter the system dynamics were analysed through bifurcations [43]. The four system behaviours represent different susceptibility of individuals to OA, which is based on the determination of parameters. Persistent inflammation is most susceptible to OA contrary to homeostasis. However, this ordinary differential equations (ODEs)-based OA inflammation model did not consider the inhibition of MMPs [30] and the reacting feedback of adipokines. In addition, the parameter estimation was based on the assumption that the parameters differ in orders of magnitude, whilst the concentration level and half-life of different mediators exhibit considerable variability [60]. This may produce biased conclusions due to the limited measurements of molecular parameters. By contrast, Rahman *et al*. [44] recently proposed a mathematical framework of cartilage degradation where the mechanical stimuli, cellular and molecular behaviours are coupled and parameters are estimated based on selected experimental data. This framework was validated by simulating the evolution of cartilaginous constituent loss according to past reported experiments. Due to the low availability of cellular and molecular data, high variability still exists in the setup of parameters that determine the outputs in the above mechanistic models. Namely, the uncertainty analysis of computational deterministic model is crucial for improving the reliability by using prospective in vivo or in vitro data.

Aforementioned mathematical models [43,44] are relatively general compared to validated models specific to cartilage lesion formation [45,47–50]. Nevertheless, the latter might not be able to unravel the mechanism of early OA inflammation since the source of injury (either mechanics or inflammation) that leads to the cartilage defects cannot be identified. In addition, none of them considers the regulatory mechanism of adipokines that are the critical mediators stimulating OA inflammation due to obesity. Based on the previous inflammation model of cartilage [43], a general five-variable mathematical model of adipokine-mediated inflammation was developed in this study and the aim was to evaluate the molecular regulations of cartilage inflammation under the reactions of adipokines.

Compared to the previous four-variable model [43], the feedback from adipokines and the inhibiting pathways of anti-inflammatory cytokines were appended to this five-variable model. BMI was introduced to measure adiposity. Meanwhile, physical activity and daily nutrition, regulating the amount of adiposity, were accounted to control the production rate of adipokines. Local sensitivity analysis and bifurcations of estimated non-dimensionalised parameters were conducted to verify this model and to evaluate the regulatory mechanism of inflammation dynamics by adipokines. In particular, the evolution of the inflammatory process was studied by implementing different physical activity interventions within the dimensionless model.

## Methods

### Regulatory network of inflammation

**Fig 1:**
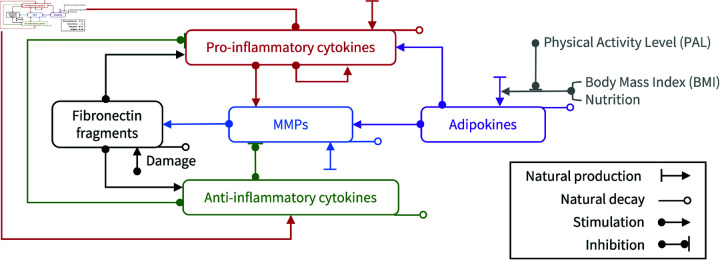
Schematic inflammatory network of cartilage including the concentrations of PICs, AICs, MMPs, adipokines and Fn-fs as denoted respectively by $P_{c},A_{c},M,A_{d},F$. Adipokines stimulate the production of PICs and MMPs as a source mediator of which concentration is regulated by adiposity. Adiposity can be varied by the system status including PAL, BMI and nutrition. Each mediator group has a rate of natural decay and the rate of production consists of the production from background, stimuli and inhibitors. Since Fn-fs are only released due to the deconstruction of tissue, the production is determined by mechanical damage and the catabolism of MMPs.

Five mediators regulating the inflammation are identified as the variables in the mathematical model. PICs and AICs, MMPs and Fn-fs interact to regulate inflammation in OA [24]. On this basis, adipokines dependent on obesity and exercise can stimulate inflammation through the concurrent upregulations of PICs and MMPs [61]. An inflammatory network of five variables ($P_{c},A_{c},M,A_{d},F$) was constructed to represent stimulation, inhibition, natural production and decay of each of the variables (Fig 1).

### Enzyme kinetics

Hill functions [62] were used to simulate the enzyme kinetics that is the reactive production rate of one mediator regulated by other mediators and to describe the nonlinear reduction of adipokines due to nutrition and physical activity:

$$H^{stimulus}\left(var_{s}\right)=MPR\times \frac{var_{s}^{n}\left(t\right)}{SC^{n}+var_{s}^{n}\left(t\right)}$$ (1)

$$H^{inhibitor}\left(var_{i}\right)=TPR\times \frac{SC^{n}}{SC^{n}+var_{i}^{n}\left(t\right)}$$ (2)

where *MPR* is the maximum driven production rate of the mediator regulated by the stimulatory mediator $var_{s}$, and *TPR* is the total production rate in the pathways inhibited by the mediator $var_{i}$. The saturation constant is *SC*, which is the concentration of the regulated mediator. At concentration *SC*, the production rates are half of *MPR* and *TPR* due to the saturated effects. The Hill coefficient *n* describes the cooperativity of the targeted mediator. Namely, the change of the slope for the increase or decrease of the production rate can be altered by tuning the Hill coefficient. In inflammatory regulations, each pathway normally involves multiple receptors; therefore, the value of Hill coefficient might equal or exceed 2. To reduce the complexity, it is assumed that *n* equals 2 in this model as the system behaviours exhibit similarity when Hill coefficients are greater than 2 [43]. The inhibition is essentially signalled by the binding between ligands and cellular receptors. Since the receptors of MMPs, PICs and AICs are distinct [30,33], the inhibition of AICs is applicable to all production terms of each signalling pathway regardless of cell sources. In the application of Hill functions, *TPR* is accordingly the total production rate by summing up a natural production rate with the driven production rates, as shown in Eqs (3) and (5).

### Mathematical formulation

The production of PICs is inherently at a low level to facilitate normal ECM remodelling, leading to a term of natural production. This metabolic activity is rigorously controlled by the regulated production and the natural decay under the healthy homeostatic background. In this model, the secretion of PICs is stimulated by itself, adipokines and Fn-fs but inhibited by AICs. The production terms are additive as the activated receptors are different in those pathways [30]. Thus, the production of PICs is formulated in Eq (3).

$$\frac{dP_{c}\left(t\right)}{dt}=[C_{0}+C_{1}\cdot \frac{P_{c}^{n}\left(t\right)}{C_{2}^{n}+P_{c}^{n}\left(t\right)}+C_{3}\cdot \frac{A_{d}^{n}\left(t\right)}{C_{4}^{n}+A_{d}^{n}\left(t\right)}\;\;+C_{5}\cdot \frac{F^{n}\left(t\right)}{C_{6}^{n}+F^{n}\left(t\right)}]\cdot \frac{C_{7}^{n}}{C_{7}^{n}+A_{c}^{n}\left(t\right)}-D_{1}\cdot P_{c}\left(t\right)$$ (3)

In turn, AICs are produced due to the increase of PICs and Fn-fs in order to counteract inflammation and maintain the homeostasis of tissue remodelling. Since the release of AICs is mainly determined by cells from the activation of immune system, there is no natural production term compared to PICs that are secreted by immune cells as well as local cells in cartilage [30]:

$$\frac{dA_{c}\left(t\right)}{dt}=C_{8}\cdot \frac{P_{c}^{n}\left(t\right)}{C_{9}^{n}+P_{c}^{n}\left(t\right)}+C_{10}\cdot \frac{F^{n}\left(t\right)}{C_{11}^{n}+F^{n}\left(t\right)}-D_{2}\cdot A_{c}\left(t\right)$$ (4)

As the enzyme degrades the cartilage tissue, MMPs are upregulated by the signalling of PICs and adipokines with a low natural production, which could be inhibited by AICs to reduce the catabolic effects of MMPs [33]. Similar to PICs, the inhibition of AICs is applied to all production terms as shown in Eq (5).

$$\frac{dM\left(t\right)}{dt}=[C_{12}+C_{13}\cdot \frac{P_{c}^{n}\left(t\right)}{C_{14}^{n}+P_{c}^{n}\left(t\right)}+C_{15}\cdot \frac{A_{d}^{n}\left(t\right)}{C_{16}^{n}+A_{d}^{n}\left(t\right)}]\cdot \frac{C_{17}^{n}}{C_{17}^{n}+A_{c}^{n}\left(t\right)}-D_{3}\cdot M\left(t\right)$$ (5)

Adipokines are mainly released from adipocytes, so the number and size of adipose cells presumably determine the production of adipokines. In general, an individual normally maintains a constant number of fat cells regardless of the weight loss since adolescence but the size increases due to the dramatic weight gain [63]. Proper physical activity intervention can induce the reduction of adiposity, whereas there is no strong evidence indicating that the number of adipocytes concomitantly decreases [64]. The present consensus is that physical activity is able to reduce the size of fat cells as the mass of adipose tissue [20,64]. This leads to a background production term due to the adipocyte number, a decay term dependent on the concentration and a variable production term determined by BMI, nutrition and PAL:

$$\frac{dA_{d}\left(t\right)}{dt}=C_{18}+[C_{19}\cdot \;f\left(BMI^{meas}\right)\cdot \frac{DailyCal}{BMR\cdot PAL}]\cdot \frac{C_{20}^{nex}}{C_{20}^{nex}+A_{d}^{nex}\left(t\right)}-D_{4}\cdot A_{d}\left(t\right)$$ (6)

where *DailyCal* is the daily intake of calories, *BMR* is the value of basal metabolic rate (BMR) and *PAL* is the level of physical activity. The function of BMI, $f\left(BMI^{meas}\right)$, is built to assume the adipokine production due to adiposity level:

$$f\left(BMI^{meas}\right)=\frac{BMI^{meas}}{BMI^{std}}$$ (7)

where ${\text{BMI}}^{\text{std}}$ is the standard value of BMI at which the inflammation system is at the normal level of body weight. Although obesity is classified by incremental ranges of BMI in Table 1 [8], a specific value of 25 is selected in order to measure the assumed proportional correlation [35] between the adipokine production and the measured BMI value, ${\text{BMI}}^{\text{meas}}$.

**Table 1:** The classification of the obesity level using BMI [8].

Level of obesity	BMI ($\text{kg}/{\text{m}}^{2}$ )
Nutritional deficiency (ND)	<18.5
Normal weight (NW)	18.5–24.9
Overweight (OW)	25–29.9
Obesity (O)	⩾30
Extreme obesity (EO)	⩾40

**Fig 2:**
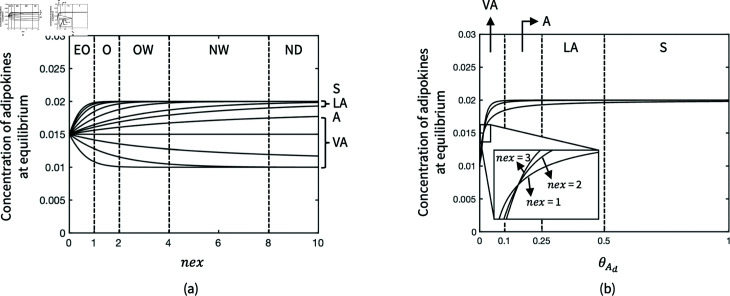
The sensitivity of (a) nex and (b) $\theta_{A_{d}}$ to the adipokine concentration at equilibrium. *nex* is numerically correlated to obesity level so it measures the sensitivity of adipokine reduction due to different PALs at a certain level of obesity (EO: Extreme obesity; O: Obesity; OW: Overweight; NW: Normal weight; ND: Nutritional deficiency). $\theta_{A_{d}}$ is the dimensionless coefficient that determines the amount of adipokine reduction due to PAL (S: Sedentary; LA: Low active; A: Active; VA: Very active).

Since PAL is measured by the proportion of total daily energy expenditure (TEE) to BMR [65], the nutrient term is described as the fraction of the daily intake of calories (*DailyCal*) to the total metabolic rate determined by BMR and PAL. The adipokine production decided by BMI and nutrition is altogether reduced by PAL through the Hill function. To distinguish the coefficient that modulates the effects of physical activity from other Hill coefficients that represent the order of enzyme kinetics, *nex* is used in Eq (6). The coefficient *nex* and saturation constant *C*_20_ are controlled by ${\text{BMI}}^{\text{meas}}$ and *PAL* respectively to simulate the nonlinear effects of different exercise levels (Table 2) on the adiposity reduction. As *nex* increases, the reduction gradient of adipokine production due to PAL ascends, namely, the increase of PAL is effective for reducing the production of adipokines when adipokines are nearly or over saturated. Meanwhile, *C*_20_ determines the saturation of adipokines where PAL reacts on the loss of adiposity. The sensitivity of *nex* and *C*_20_ and their correlation with ${\text{BMI}}^{\text{meas}}$ and *PAL* is analysed in a dimensionless manner (Fig 2).

**Table 2:** The category of PAL [65].

Level of physical activity	PAL value
Sedentary (S)	1–1.39
Low active (LA)	1.4–1.59
Active (A)	1.6–1.89
Very active (VA)	1.9–2.5

Since the level of damage can also induce inflammation, Fn-fs, the biomarker from degraded cartilage tissue upregulates both PICs and AICs [38], is included in this model to measure the damage due to inflammation $\left(C_{21}\cdot M\right)$ and excessive mechanical loading $\left(C_{22}\right)$:

$$\frac{dF\left(t\right)}{dt}=C_{21}\cdot M\left(t\right)+C_{22}-D_{5}\cdot F\left(t\right)$$ (8)

### Model nondimensionalisation and parameter estimation

For the mathematical analysis, the model is non-dimensionalised by the following scaling:


$$P_{c}\left(t\right)=C_{2}\cdot \tilde{P_{c}\left(t\right)}$$

$$A_{c}\left(t\right)=C_{7}\cdot \tilde{A_{c}\left(t\right)}\;M\left(t\right)=\frac{C_{6}D_{2}}{C_{21}}\cdot \tilde{M\left(t\right)}\;A_{d}\left(t\right)=C_{4}\cdot \tilde{A_{d}\left(t\right)}\;F\left(t\right)=C_{6}\cdot \tilde{F\left(t\right)}\;t=\frac{\tilde{t}}{D_{2}}\;$$ (9)

where the concentrations of PICs, AICs, adipokines and Fn-fs are non-dimensionalised by the corresponding saturation constants, and the time is scaled by the decay rate of AICs for the convenience of model verification. The concentration of MMPs is scaled to reduce the Fn-fs production.

The non-dimensionalised model is shown in Eqs (10) to (14) and the estimated parameters are indicated in Table 3.

**Table 3:** The estimated dimensionless parameters of the inflammation model.

Parameters	Description	Value	Reference
$\alpha_{BP_{c}}$, $\alpha_{BM}$	Natural production rate	0.01	[43]
$\beta_{P_{c}P_{c}}$, $\beta_{A_{d}P_{c}}$, $\beta_{FP_{c}}$, $\beta_{P_{c}A_{c}}$, $\beta_{FA_{c}}$, $\beta_{P_{c}M}$, $\beta_{A_{d}M}$	Stimulated production rate	10	[43]
$\theta_{P_{c}A_{c}}$, $\theta_{FA_{c}}$, $\theta_{P_{c}M}$, $\theta_{A_{d}M}$, $\theta_{A_{c}M}$	Saturation constant at which the capability of stimulation or inhibition is half of maximum	1	[43]
$\mu_{NA_{d}}$	The background production rate of adipokines due to the number of adipocytes	0.01	Estimated
$\mu_{SA_{d}}$	The background production rate of adipokines due to the size of adipocytes	0.01	Estimated
$\gamma_{P_{c}}$, $\gamma_{M}$, $\gamma_{A_{d}}$, $\gamma_{F}$	Clearance rate	1	[43]
*Damage*	The damage level of cartilage	0	[43]
${\text{BMI}}^{\text{std}}$	Standard value of BMI	25	[8]
$\frac{DailyCal}{BMR}$	Nutrition factor	1	Estimated
$\theta_{A_{d}}$, *nex*	Coefficients in the inhibition of adipokine production due to exercise	Dependent on PAL and ${\text{BMI}}^{\text{meas}}$	Estimated
*n*	Hill coefficient	2	[43]

$$\frac{d\tilde{P_{c}}\left(\tilde{t}\right)}{d\tilde{t}}=[\alpha_{BP_{c}}+\beta_{P_{c}P_{c}}\cdot \frac{{\tilde{P_{c}}}^{n}\left(\tilde{t}\right)}{1+{\tilde{P_{c}}}^{n}\left(\tilde{t}\right)}+\beta_{A_{d}P_{c}}\cdot \frac{{\tilde{A_{d}}}^{n}\left(\tilde{t}\right)}{1+{\tilde{A_{d}}}^{n}\left(\tilde{t}\right)}\;\;+\beta_{FP_{c}}\cdot \frac{{\tilde{F}}^{n}\left(\tilde{t}\right)}{1+{\tilde{F}}^{n}\left(\tilde{t}\right)}]\cdot \frac{1}{1+{\tilde{A_{c}}}^{n}\left(\tilde{t}\right)}-\gamma_{P_{c}}\cdot \tilde{P_{c}}\left(\tilde{t}\right)$$ (10)

$$\frac{d\tilde{A_{c}}\left(\tilde{t}\right)}{d\tilde{t}}=\beta_{P_{c}A_{c}}\cdot \frac{{\tilde{P_{c}}}^{n}\left(\tilde{t}\right)}{\theta_{P_{c}A_{c}}^{n}+{\tilde{P_{c}}}^{n}\left(\tilde{t}\right)}+\beta_{FA_{c}}\cdot \frac{{\tilde{F}}^{n}\left(\tilde{t}\right)}{\theta_{FA_{c}}^{n}+{\tilde{F}}^{n}\left(\tilde{t}\right)}-\tilde{A_{c}}\left(\tilde{t}\right)$$ (11)

$$\frac{d\tilde{M}\left(\tilde{t}\right)}{d\tilde{t}}=[\alpha_{BM}+\beta_{P_{c}M}\cdot \frac{{\tilde{P_{c}}}^{n}\left(\tilde{t}\right)}{\theta_{P_{c}M}^{n}+{\tilde{P_{c}}}^{n}\left(\tilde{t}\right)}\;\;+\beta_{A_{d}M}\cdot \frac{{\tilde{A_{d}}}^{n}\left(\tilde{t}\right)}{\theta_{A_{d}M}^{n}+{\tilde{A_{d}}}^{n}\left(\tilde{t}\right)}]\cdot \frac{\theta_{A_{c}M}^{n}}{\theta_{A_{c}M}^{n}+{\tilde{A_{c}}}^{n}\left(\tilde{t}\right)}-\gamma_{M}\cdot \tilde{M}\left(\tilde{t}\right)$$ (12)

$$\frac{d\tilde{A_{d}}\left(\tilde{t}\right)}{d\tilde{t}}=\mu_{NA_{d}}+[\mu_{SA_{d}}\cdot \;f\left(BMI^{meas}\right)\cdot \frac{DailyCal}{BMR\cdot PAL}]\cdot \frac{\theta_{A_{d}}^{nex}}{\theta_{A_{d}}^{nex}+{\tilde{A_{d}}}^{nex}\left(\tilde{t}\right)}\;\;-\gamma_{A_{d}}\cdot \tilde{A_{d}}\left(\tilde{t}\right)$$ (13)

$$\frac{d\tilde{F}\left(\tilde{t}\right)}{d\tilde{t}}=\tilde{M}\left(\tilde{t}\right)+Damage-\gamma_{F}\cdot \tilde{F}\left(\tilde{t}\right)$$ (14)

### Sensitivity analysis

The dependence of the inflammation behaviours on the isolated non-dimensionalised parameters in this model was analysed through Local Sensitivity Analysis (LSA), which was verified by comparing with Baker *et al*. [43].

The LSA was implemented with the maximum ±30% perturbation of each parameter [43] for the comparison:

$$S_{K}^{M}=\frac{\delta M\left(\omega \right)╱M\left(\omega \right)}{\delta K╱K}$$ (15)

where *K* is the reference parameter that was perturbed and M(ω) is the measured response based on the system output ω. The responses include the mean steady concentration at the healthy state and inflamed limit cycles, the amplitude and period in limit cycles of inflammation [66]. Correspondingly, δK is the perturbation of the parameter *K* and its effect on the measured response is δM(ω). In particular, parameters were randomly perturbed 1,000 times [43] in the range of [−0.3,+0.3] that is uniformly distributed so that the relative sensitivity can be represented by the Interquartile Range (IQR) of the responses from 1,000 perturbations.

The bifurcations of sensitive parameters were analysed to explore the dynamics of inflammation with obesity and compared to Baker *et al*. [43] for the verification of the five-variable model and MATLAB scripts for bifurcation analysis. In particular, the values of the parameters estimated in Table 3 are also the baseline for bifurcation analysis. The concentration of PICs measures the inflammation level at steady states to present the transitions of system dynamics. The steady states were accordingly given by the simultaneous equations (S7 Eqs) derived for calculating equilibrium solutions, in conjunction with the phase plane of the system under baseline parameters (S8 Fig). The stability of local behaviour was examined by the application of linearisation to the obtained equilibrium solutions. Due to the complex nonlinearity of this model, the Jacobian of this system and the corresponding eigenvalues were numerically computed for the linear approximation in bifurcation analysis.

**Fig 3:**
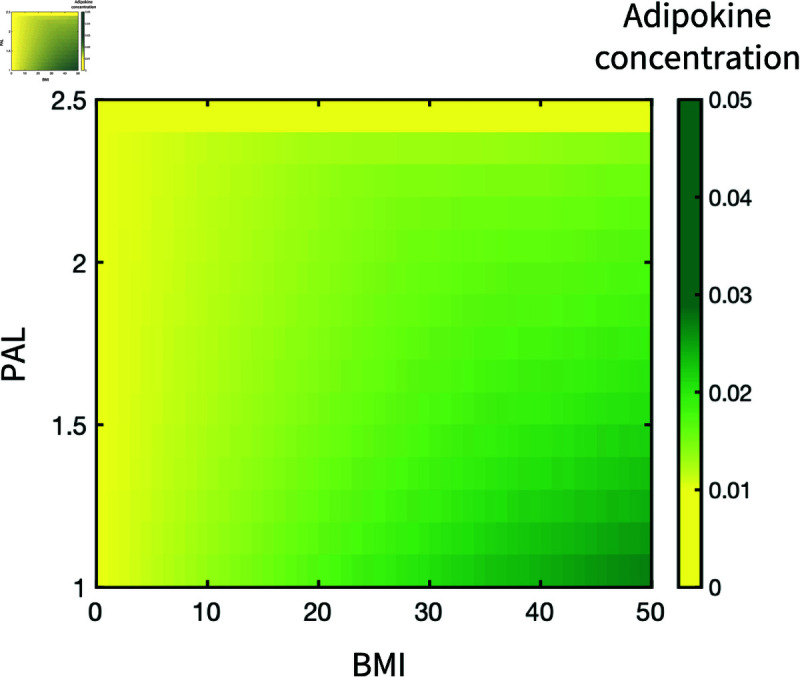
The equilibrium adipokine concentration against PAL and BMI.

In addition, the sensitivity of adiposity regulation by PAL was evaluated by measuring the variation of adipokine concentration at equilibrium when tuning *nex* and $\theta_{A_{d}}$. The categories of obesity level (Table 1) and PAL (Table 2) are used to define the different gradients of the concentration variations (Fig 2). Given the sensitivity, two piecewise continuous functions, Eqs (16) and (17), are provided with the boundary parameters to estimate *nex* and $\theta_{A_{d}}$ based on different combinations of BMI and PAL. It is assumed that the reduction of adipokines is approximately linear to a certain PAL range when the obesity level remains the same, leading to a linear correlation between the non-dimensionalised parameters and inputs of BMI or PAL when they are in the same category. Fig 3 illustrates the concentration of adipokines at equilibrium with different values of BMI and PAL, which are governed by Eqs (16) and (17).

$$nex=\left\{ \begin{array}{cc} B_{ND}-BMI^{meas}\cdot \frac{B_{ND}-B_{NW}}{R_{ND}-R_{ND_{min}}}, & if\;R_{ND_{min}}<BMI^{meas}<R_{ND}\\ B_{NW}-\left(BMI^{meas}-R_{ND}\right)\cdot \frac{B_{NW}-B_{OW}}{R_{NW}-R_{ND}}, & if\;R_{ND}⩽BMI^{meas}⩽R_{NW}\\ B_{OW}-\left(BMI^{meas}-R_{NW}\right)\cdot \frac{B_{OW}-B_{O}}{R_{OW}-R_{NW}}, & if\;R_{NW}<BMI^{meas}⩽R_{OW}\\ B_{O}-\left(BMI^{meas}-R_{OW}\right)\cdot \frac{B_{O}-B_{EO}}{R_{O}-R_{OW}}, & if\;R_{OW}<BMI^{meas}<R_{O}\\ \frac{R_{O}}{BMI^{meas}}, & if\;BMI^{meas}⩾R_{O} \end{array}\right.$$ (16)

$$\theta_{A_{d}}=\left\{\begin{array}{cc} B_{S}-\left(PAL-R_{S_{min}}\right)\cdot \frac{B_{S}-B_{LA}}{R_{S}-R_{S_{min}}}, & if\;R_{S_{min}}⩽PAL⩽R_{S}\\ B_{LA}-\left(PAL-R_{S}\right)\cdot \frac{B_{LA}-B_{A}}{R_{LA}-R_{S}}, & if\;R_{S}<PAL⩽R_{LA}\\ B_{A}-\left(PAL-R_{LA}\right)\cdot \frac{B_{A}-B_{VA}}{R_{A}-R_{LA}}, & if\;R_{LA}<PAL⩽R_{A}\\ B_{VA}-\left(PAL-R_{A}\right)\cdot \frac{B_{VA}}{R_{VA}-R_{A}}, & if\;R_{A}<PAL⩽R_{VA} \end{array}\right\}$$ (17)

where the boundaries of *nex* and $\theta_{A_{d}}$ are estimated according to Fig 2: *B*_*ND*_ = 10, *B*_*NW*_ = 8, *B*_*OW*_ = 4, *B*_*O*_ = 2, *B*_*EO*_ = 1, *B*_*S*_ = 1, *B*_*LA*_ = 0.5, *B*_*A*_ = 0.25, $B_{VA}=0.1$. In addition, parameters of BMI and PAL range are determined by Tables 1 and 2: $R_{ND_{min}}=0$, *R*_*ND*_ = 18.5, *R*_*NW*_ = 24.9, *R*_*OW*_ = 29.9, *R*_*O*_ = 40, $R_{S_{min}}=1$, *R*_*S*_ = 1.39, *R*_*LA*_ = 1.59, *R*_*A*_ = 1.89, $R_{VA}=2.5$.

### Simulation of inflammatory activities

The inflammatory activities were simulated by solving the governing ODEs in MATLAB (R2022b, The Math Works, Inc., Natick, MA, USA) under the estimated parameters to evaluate the effects of obesity and PAL. The MATLAB function ode45 was used as the solver of the explicit Runge-Kutta method with the relative tolerance of 0.001 and the absolute tolerance of $1\;\times \;10^{-6}$ for simulations. Due to the lack of experimental data, the time scale of the model is governed by the decay rate of each variable. However, the uncertainty of decay rates remains in either the same or different mediator groups. In order to analyse the evolution of OA inflammation when the intervention of physical activity was applied, the dimensionless concentration of each mediator was measured within 100 dimensionless time units. Particularly, the level of cytokines reflects the inflammation process, and the concentration of Fn-fs measures the damage due to the combined effects of inflammation and mechanics.

The minimum values of mechanical damage leading to inflammation were compared over the inflammation system with different obesity levels. In addition, a sudden damage (*Damage* = 0.005) due to mechanics (injury) was applied to the model at time point 20, and PAL increases from a sedentary level (*PAL* = 1) to a low active level (*PAL* = 1.5) to simulate physical therapy. The input characteristics of this computational subject also include BMI (*BMI*^*meas*^ = 30) and daily nutrition (DailyCal╱BMR=1) to study the effects of PAL on the inflammatory process of an obese subject.

## Results

### Model verification

**Fig 4:**
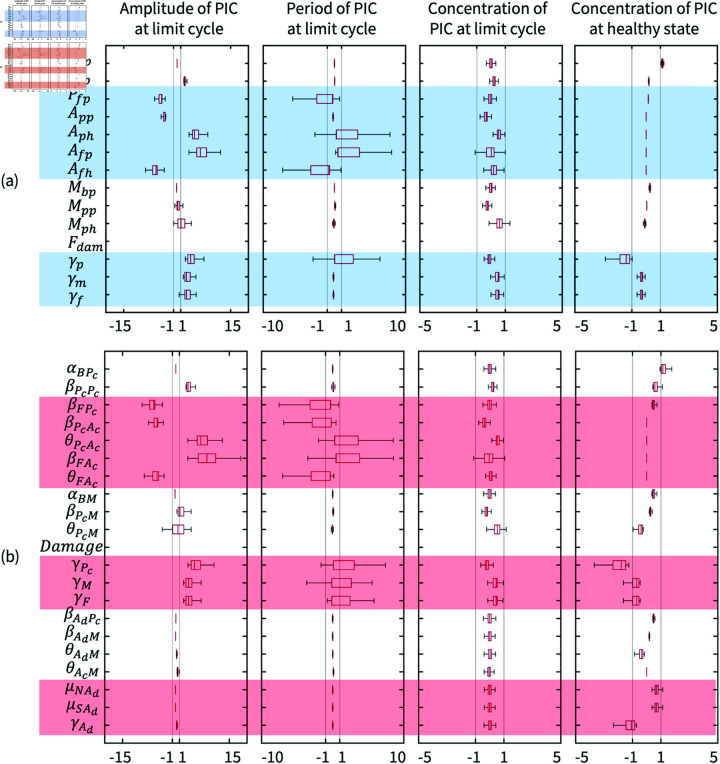
The boxplots of the LSA for dimensionless parameters in (a) Baker’s model [43] and (b) the adipokine-mediated inflammation model. The values of datasets represent the percentages of change in the measured responses from the system outputs perturbed by 1 percent change of a specified parameter at the inflamed limit cycle and the healthy state. Parameters that result in output changes over 1 percent are highlighted in blue for (a) and in red for (b). *P*_*bp*_: The background production rate of PICs; *P*_*pp*_: The production rate of PICs driven by PICs; *P*_*fp*_: The production rate of PICs driven by Fn-fs; *A*_*pp*_: The production rate of AICs driven by PICs; *A*_*ph*_: The concentration of AICs at which the production rate of AICs driven by PICs is half of the maximum; *A*_*fp*_: The production rate of AICs driven by Fn-fs; *A*_*fh*_: The concentration of AICs at which the production rate of AICs driven by Fn-fs is half of the maximum; *M*_*bp*_: The background production rate of MMPs; *M*_*pp*_: The production rate of MMPs driven by PICs; *M*_*ph*_: The concentration of MMPs at which the production rate of MMPs driven by PICs is half of the maximum; *F*_*dam*_: Mechanical damage parameter; $\gamma_{p}$: Decay rate of PICs; $\gamma_{m}$: Decay rate of MMPs; $\gamma_{f}$: Decay rate of Fn-fs.

In order to verify the model of adipokine-mediated inflammation and analyse the variations in system behaviours due to adipokine, LSA and bifurcation diagrams of sensitive parameters were compared between Baker’s model [43] and this five-variable model. The IQR of perturbed system outputs (Fig 4) illustrates that the parameters relevant to adipokine production and its regulation are insensitive to the inflammation state. Instead, the PIC natural production ($\alpha_{BP_{c}}$) and clearance rates ($\gamma_{P_{c}}$ and $\gamma_{A_{d}}$) of PICs and adipokines slightly affect the cytokine concentration in the healthy state. For the inflammatory oscillation, the amplitude of cytokine concentration is readily perturbed when parameters of inhibition ($\theta_{P_{c}A_{c}}$ and $\theta_{FA_{c}}$) and stimulated production ($\beta_{FP_{c}},\beta_{P_{c}A_{c}}$ and $\beta_{FA_{c}}$) are varied, and the relative sensitivity is similar in the inflammatory amplitude in the five-variable model and Baker’s model. However, the introduction of adipokines sensitises $\beta_{P_{c}A_{c}},\gamma_{M}$ and $\gamma_{F}$ in the response of the inflammatory period. This suggests that obesity affects the OA inflammatory process by changing the sensitivity of other parameters to the system period in limit cycle, namely, parameters of adipokines have impacts on the inflammation dynamics and they can slightly induce the release of cytokines at the healthy state.

Bifurcations of the common parameters were compared between Baker’s model [43] and this five-variable model in S1 Fig, S2 Fig, S3 Fig and S4 Fig. The addition of adipokines reduces the bistability and the inflamed limit cycle of the inflammation system for a range of values of most parameters, which results in the system staying in persistent inflammation in a wider range of parameter perturbations.

### Dynamics of the adipokine-mediated inflammation model

The types of system dynamics reflected in the bifurcation diagrams of this study are summarised in Table 4. Tristable behaviours with five steady states were found when varying parameters in the study by Baker *et al*. [43]. It is important to note that the variation of parameters might deviate from the assumptions of parameter estimation, although system tristability is potentially one of the mathematical solutions to the inflammation model. According to the intersection of nullclines in the phase plane diagram of this model (S8 Fig), five steady states also exist when artificially varying the parameters. However, in order to prevent numerical artefacts due to parameter estimation, the analysis of the system dynamics focuses on the effects of obesity when the reference parameter set is maintained at baseline. The exhaustive estimation was not implemented for the parameters irrelevant to adipokines. Representative steady states (health, oscillatory inflammation and steady inflammation) were mainly found in the state transitions resulting from the variability in parameters governing adipokine regulation.

**Table 4:** The types of dynamics presented in bifurcations. S: Quiescent stable state; L: Limit cycle; S in superscript: Stable; U in superscript: Unstable.

Dynamics type	The number of steady state	Stability	The type of steady state
*I*_*monostable*_	$S_{0}^{S}$	Monostable	Persistent steady inflammation
*II*_*monostable*_	$L_{0}^{S}$	Monostable	Persistent oscillatory inflammation
*III*_*bistable*_	$S_{0}^{S},S_{1}^{U},L_{0}^{S}$	Bistable	Health, oscillatory inflammation
${IV}_{bistable}$	$S_{0}^{S},S_{1}^{U},S_{2}^{S}$	Bistable	Health, steady inflammation
$V_{monostable}$	$S_{0}^{S}$	Monostable	Health
${VI}_{monostable}$	$S_{0}^{S},S_{1}^{U},S_{2}^{U}$	Monostable	Health

**Fig 5:**
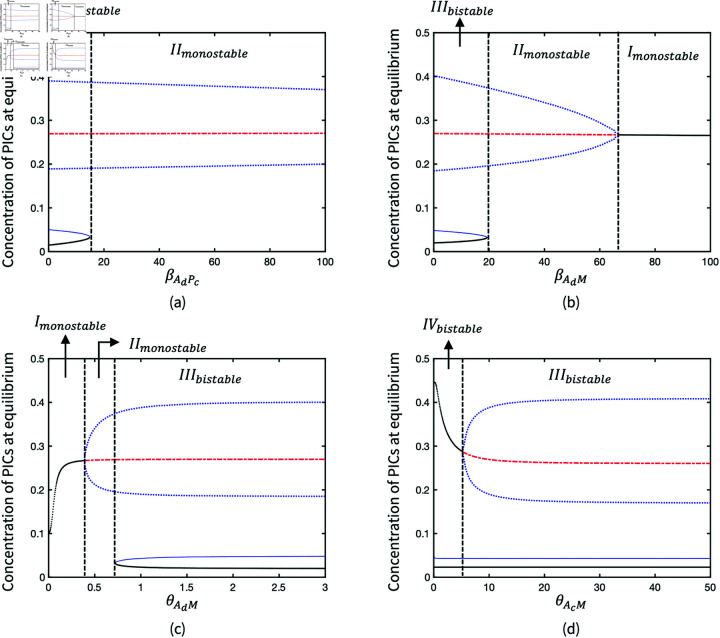
Bifurcation diagrams of parameters in the production of PICs and MMPs: (a) Production rate of PICs driven by adipokines; (b) Production rate of MMPs driven by adipokines; (c) Saturation rate in the stimulation of MMPs by adipokines; (d) Saturation rate in the inhibition of MMPs by AICs. Solid black lines where the pro-inflammatory is at a higher level represent the inflamed states; solid black lines where the pro-inflammatory is at a lower level represent the healthy states; dash blue lines represent the unstable states; solid blue lines represent unstable states; The scatter of blue points represents the average maximum and minimum concentration in the oscillated limit cycle.

There are three parameters $\beta_{A_{d}P_{c}},\beta_{A_{d}M}$ and $\theta_{A_{d}M}$, associated with adipokine level to regulate the production of PICs and MMPs. Their bifurcations (Figs 5a to 5c) indicate that the high production rates of both PICs ($\beta_{A_{d}P_{c}}$) and MMPs ($\beta_{A_{d}M}$ and $\theta_{A_{d}M}$) driven by adipokines lead to the loss of healthy state. Unlike the inflamed limit cycle due to the high value of $\beta_{A_{d}P_{c}}$, the increase of $\beta_{A_{d}M}$ and the reduction of $\theta_{A_{d}M}$ can result in the quiescent steady inflammation state. This suggests that the stimulation of PICs is not as sensitive as MMPs due to adipokines. Due to the nondimensionalisation of parameters, $\theta_{A_{c}M}$ represents the weight of two inhibition pathways, the inhibitions of PICs and MMPs by AICs. When $\theta_{A_{c}M}$ decreases, the weight of MMPs inhibition decreases and the system stays bistable but the inflammatory process tends to be stable rather than oscillatory (Fig 5d).

**Fig 6:**
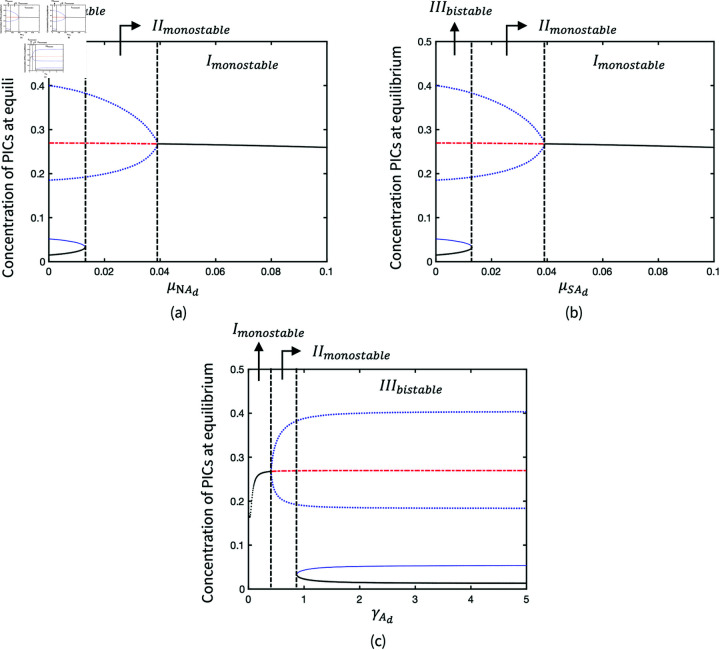
Bifurcation diagrams of parameters in the production of adipokines: (a) Production rate of adipokines due to the number of adipocytes; (b) Production rate of adipokines due to the size of adipocytes; (c) Decay rate of adipokines.

Fig 6 illustrates the variations of system dynamic behaviours due to the changes of parameters ($\mu_{NA_{d}}$, $\mu_{SA_{d}}$ and $\gamma_{A_{d}}$) in adipokine production. Bifurcations of $\mu_{NA_{d}}$ and $\mu_{SA_{d}}$ (Figs 6a and 6b) are similar as the weights of adipocyte number and size represented by those two parameters of the adipokine production are initially equal in the production of adipokines. A threshold of nearly 0.02 for the adipokine production rates ($\mu_{NA_{d}}$ and $\mu_{SA_{d}}$) indicates a transition from *III*_*bistable*_ to *II*_*monostable*_ (Figs 6a and 6b), resulting in persistent inflammation and the loss of quiescence healthy state. In turn, the increase of adipokine decay rate ($\gamma_{A_{d}}$) turns the monostable system of inflammation to bistable system so that the healthy state returns (Fig 6c).

**Fig 7:**
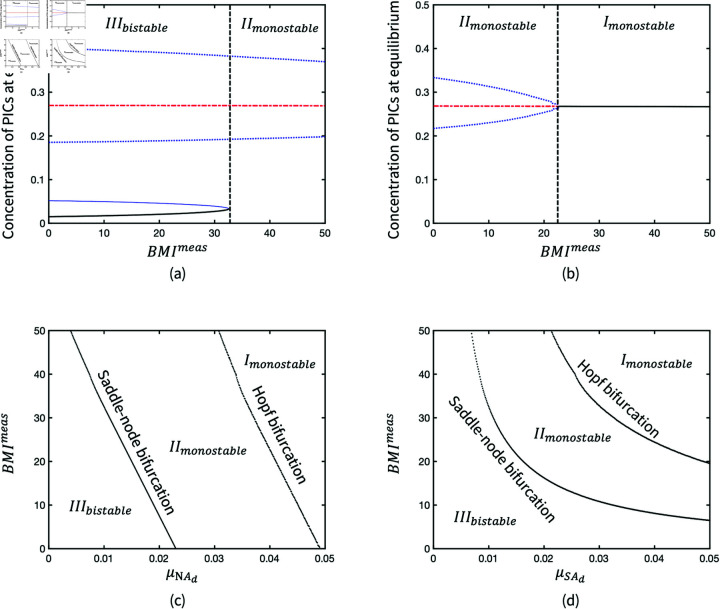
Bifurcation diagrams of ${\text{BMI}}^{\text{meas}}$ where (a) $\mu_{NA_{d}}=0.01$, $\mu_{SA_{d}}=0.01$; (b) $\mu_{NA_{d}}=0.04$, $\mu_{SA_{d}}=0.01$, and codimension-2 bifurcations of (c) $\mu_{NA_{d}}$ and ${\text{BMI}}^{\text{meas}}$; (d) $\mu_{SA_{d}}$ and ${\text{BMI}}^{\text{meas}}$

${\text{BMI}}^{\text{meas}}$ is an input parameter that modulates the weight of the adipokine production term associated to adipocyte size, hence its impact on the system behaviour is similar to $\mu_{NA_{d}}$ and $\mu_{SA_{d}}$. A threshold of BMI that causes persistent inflammation is found at approximately 33 in Fig 7a when the parameters are at baseline, where $\mu_{NA_{d}}=0.01$ and $\mu_{SA_{d}}=0.01$. High BMI can reduce the bistability of the inflammation system so that it remains in a monostable inflamed limit cycle. The monostability of inflammation results in an inability of the system to return to a healthy state. In addition, the BMI threshold of bifurcation is dependent on $\mu_{NA_{d}}$ and $\mu_{SA_{d}}$. Fig 7b presents the Hopf bifurcation when oscillatory inflammation turns into persistent steady inflammation as BMI increases. In the codimension-2 bifurcations of $\mu_{NA_{d}}$ and $\mu_{SA_{d}}$, the transitions of system behaviours are presented in the range of BMI between 0 and 50 (Figs 7c and 7d). The transitions reflect the susceptibility of OA inflammation in different cohorts.

**Fig 8:**
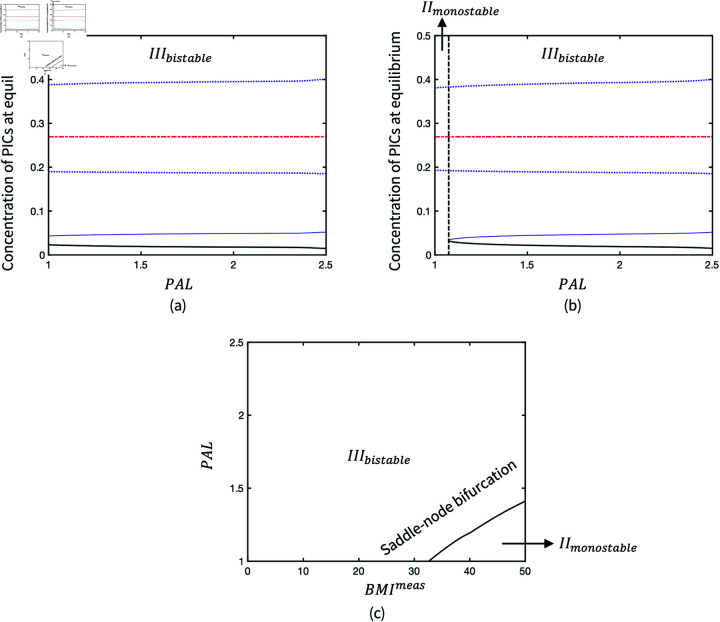
Bifurcation diagrams of PAL when (a) *BMI*^*meas*^ = 25; (b) *BMI*^*meas*^ = 35 in the production of adipokines, and (c) Codimension-2 bifurcation of ${\text{BMI}}^{\text{meas}}$ and PAL.

However, two representative bifurcation diagrams of PAL in Figs 8a and 8b illustrate that the monostability of inflammation can be changed by increasing PAL. The effectiveness of PAL also depends on the level of BMI, so a higher PAL is required to return the bistability when BMI exceeds the threshold at 33. Fig 8c shows the transition between *III*_*bistable*_ and *II*_*monostable*_ in the parameter space via the codimension-2 bifurcation of *PAL* and ${\text{BMI}}^{\text{meas}}$.

**Fig 9:**
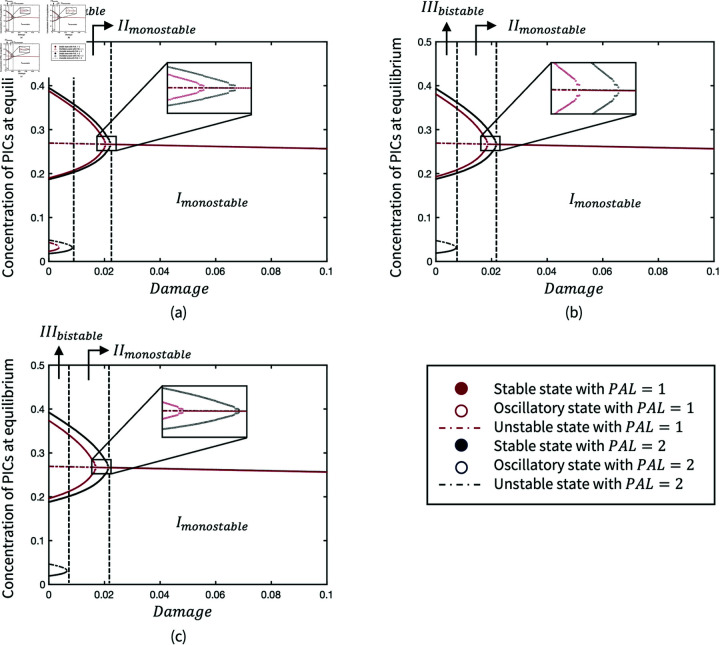
Bifurcation diagrams of Damage under different physical activity interventions when (a) *BMI*^*meas*^ = 25 ; (b) *BMI*^*meas*^ = 35; (c) *BMI*^*meas*^ = 45 in the adipokine-mediated inflammation model. The dynamics transition is presented when *PAL* = 2 as an example

In addition to BMI, mechanical damage can also lead to persistent inflammation when it exceeds a threshold, of which sensitivity is dependent on BMI as well as PAL (Fig 9). The system stays in the inflammation state and the damage is most sensitive to the system when BMI is 35 or 45 over the threshold, hence the oscillatory inflammation turns to persistent inflammation as damage is increased. Regardless of BMI, high PAL can reduce the sensitivity of damage leading to inflammation, and the reduction is more significant when BMI is higher. Namely, the minimum damage leading to a monostable inflammation state increases due to the higher PAL.

### Evolution of inflammatory activities

**Fig 10:**
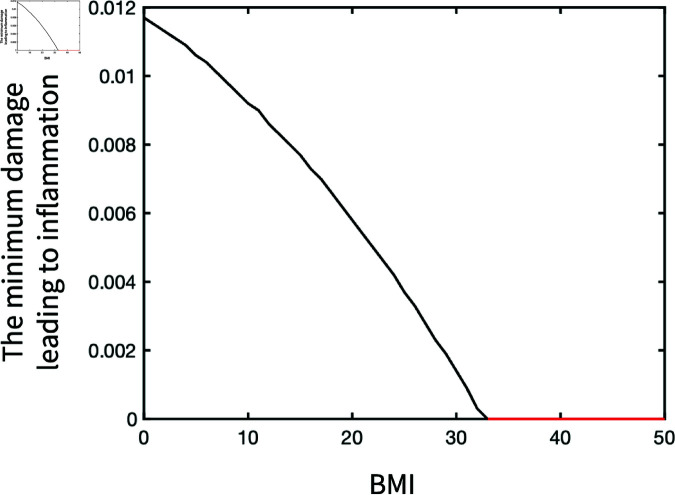
The sensitivity of the minimum damage leading to inflammation in the evolution of inflammatory activities.

**Fig 11:**
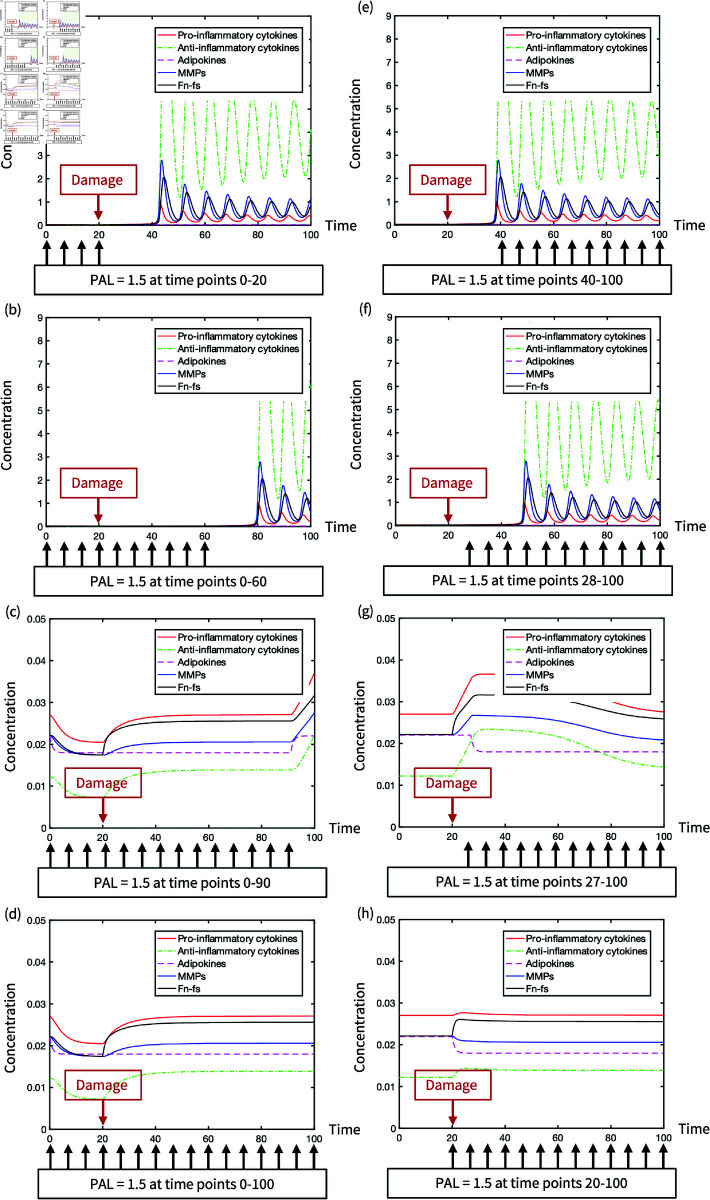
The evolution of inflammatory activities under different strategies of physical activity intervention in the non-dimensionalised model.

The nonlinear inverse correlation between BMI and the minimum mechanical damage that causes chronic inflammation is illustrated in Fig 10. The minimum damage decreases to zero when BMI is over 33 due to the transition of system behaviour to *II*_*monotable*_ from *III*_*bistable*_. In addition, the risk of inflammation significantly increases in the BMI range between 20 and 30 as the minimum damage starts to sharply decrease. However, adequate physical activity interventions that are applied before the mechanical damage can reduce the inflammatory response (Fig 11). This exhibits the significance of the timing for physical activity intervention.

The mechanical damage occurs at time point 20 when the level of inflammatory mediators is upregulated. As the inflammatory process evolves, physical activity interventions can postpone the activation of inflammation (Figs 11a to 11c) so that the system stays in the healthy state where mediator concentrations are stable. Without damage repair, the reduction of adipokines by continuous physical activity is able to prevent the system from inflammation. In turn, Figs 11e to 11h show that the system remains in oscillatory inflammation when the intervention misses an effective window period of 7 time units after damage occurs. In the time span of inflammation, the mediator concentrations fluctuate at a high level due to the nonlinear stimulations and inhibitions among inflammatory mediators. Regardless of physical activity intervention, the steady state of inflammation cannot be transited to a healthy state without any external dose to reduce the level of inflammatory mediators. Thus, the disclosure of the window period in this model emphasises the importance of a continuous physical activity intervention maintaining a low level of adipokines.

## Discussion

The first general mechanistic inflammation model of OA including the effect of obesity and exercise was developed to qualitatively analyse the dynamics of the chronic inflammatory process regulated by adipokines. This model was extended from a four-variable cartilage inflammation model [43] by formulating the major inflammatory mediators and their signalling pathways regarding OA. The assumption of molecular pathways is based on Hill functions and the law of mass action. Accordingly, the inflammatory activities can be qualitatively measured by the concentration of each mediator group within this model. The introduction of obesity and physical activity provided a novel perspective on the prevention and intervention of the chronic inflammatory process at the molecular level in OA. Obesity is measured by the number and size of adipocytes associated to the production of adipokines to present the variability of individuals susceptible to obesity. Results show that ascending adipokine production can reduce the stability of healthy state in the inflammation system via a saddle-node bifurcation so the risk of OA inflammation increases. Meanwhile, the system becomes sensitive to the parameter of damage. As an intervention to reduce adiposity, an adequate increase of PAL can return a steady state of health to significantly reduce the risk of inflammation. This is approached by the same type of saddle-node bifurcation. In the evolution of inflammatory activities, physical activity intervention can prevent the system from inflammation when inducing mechanical damage.

In addition to excessive loading, obesity leads to a aberrant level of adipokines that aggravate the inflammation during OA. Despite the protective role of adiponectin inducing AICs in OA [67], a high level of adipokines is tightly associated with the production of PICs and MMPs [35,61]. This five-variable model was verified by comparing the parameter sensitivity and bifurcations to Baker’s model [43] using the same mathematical protocol. As the signalling source of stimulating PICs and MMPs, the introduction of adipokines results in additional parameters that can alter the system dynamics. Specifically, adipokines increase the sensitivity of this adipokine-mediated inflammation system to inflammatory response as it is found that the stability at a quiescent healthy state is reduced in the bifurcations of a range of parameters. However, the local and systemic impacts of obesity are not differentiated in this model. The total inflammatory response of cartilage can stem from the local stimulation of inflammatory mediator production and systemic circulation of PICs and adipokines to the joint [16,19,42]. The systemic indicators (BMI, daily nutrition and PAL) were thereby used to measure the adipokine level regulating the total inflammation of cartilage other than specifying the source of adipokines, which provides a comprehensive impact of obesity on the inflammation dynamics of OA. The pathways of adipokines stimulating PICs and MMPs can both result in a monostable inflammation state. Particularly, the production of MMPs elevated by excessive stimulation or declined inhibition can lead the system to a steady state of inflammation without oscillation. This suggests that MMPs may preponderate in the aggravation of inflammatory activities, and the production of MMPs activated by adipokines contributes to steady inflammation, so the system firmly stays in the inflammatory activities that deteriorate cartilage tissue during OA progression.

Oscillatory inflamed limit cycle represents the early stage of OA where the high levels of cytokines, MMPs or Fn-fs are intermittent [43]. It has been reported that desultory pain of the joint occurs in individuals with early OA [68] in spite of the lack of a standard to ascertain the role of biomarkers in diagnosing the early OA stage [69–71]. Pathophysiologically, AICs are recruited and chondrocytes are active to repair the failure tissue at the early OA stage [72] when interactions of inflammatory mediators can disrupt the anabolic activities. Since the damage caused by the catabolism of inflammation or excessive mechanical loadings accumulates in OA progression [24], the inability to reduce inflammation and the release of damaging products lead to steady inflammation without oscillation. Nevertheless, the bifurcations of adipokine production parameters indicate that a significantly high level of adipokines can also cause steady inflammation when the parameter of mechanical damage is zero, which can be also seen in the bifurcations of other parameters that can upregulate the production of PICs and MMPs. This suggests that the tissue degraded by the overwhelming chronic inflammation prompts the OA process regardless of mechanical damage. In fact, progressive structural degeneration of the cartilage inevitably causes mechanical defects that can further intensify inflammation due to the abnormal biomechanical behaviours within the entire joint [73]. Thus, the identification of reasons for tissue rupture might contribute to diagnosing early OA. However, this identification is challenging since the reason might not be independent due to the combinations of multiple factors such as low tissue turnover, structural changes of tissue and potent inflammatory activities [21,74].

In this model, the mechanical impact of obesity is not specified by BMI, though the parameter of damage denotes the mechanical response on the tissue due to excessive loadings. The bifurcations of BMI and mechanical damage illustrate that a high BMI can change the sensitivity of mechanical damage to the inflammation by reducing the bistability of this system, hence the system is more susceptible to OA inflammation. Likewise, the sensitivity of minimum mechanical damage causing inflammation risk is exponentially associated with BMI. This is consistent with Berenbaum and Sellam [75] who report that the likelihood of OA onset rises by 15% with each additional unit as BMI exceeds 27. Nevertheless, the risk of OA is the concurrent outcome of the metabolic and mechanical response and the amount of injury due to high BMI is not accounted for within this model.

Body weight is one of variables in the calculation of BMI and it is reported that PAL can reduce fat mass and the correlation between PAL and body weight is weak [19,20]. Hence the application of PAL, as an interventive status in this model, essentially reduces the production of adipokines rather than body weight to alter the system dynamics. The stability of healthy state returns when applying adequate PAL if BMI is high in the bifurcations of PAL and mechanical damage, so the risk of evolving into inflammation and inflammatory damage can be decreased. However, external mechanical damage results in a window period of reducing inflammation risk by PAL. The window period was found to be 7 time units, of which the dimension is dependent on the decay rates of mediators. Outside of the window period, the intervention of PAL can only postpone inflammatory activities before the system evolves into inflammation. Accordingly, physical activity might not be able to reduce the inflammation directly, instead the susceptibility of OA inflammation can be altered and other therapeutic treatments can be more effective to control inflammatory progress. Shumnalieva *et al*. [5] highlighted that the combination of physical activity and dietary intervention could improve the success rate of pharmacological therapies for the phenotypes of OA associated with obesity, though the increase of mitochondrial biogenesis that inhibits cytokine production [22] due to above interventions is not considered. In this model, physical activity intervention reduces adipokine production to inhibit cytokine production, whose role is similar to the increased mitochondrial biogenesis in the inflammatory process. Therefore, the influence of physical activity is to regulate the dynamics of the inflammation system at the molecular level.

Whereas this general model includes the regulatory pathways of adipokines in OA inflammation, the lack of biological and clinical data raises challenges on its validation. Moreover, inflammatory mediators are categorised into different functional groups so there might be no specific data for the parameter estimation. As the consideration of model complexity and computing demands, the general classification of mediator groups is efficacious to simulate the inflammatory activities [58]. The association between inflammation and structural changes of tissue can be considered by including the spatial dimension. However, this ODE-based model can reduce computing complexity and focus on the time-dependent molecular interactions in inflammation. Molecules might derive from the cross-talk at different spatial levels, which is implicitly reflected in the outcomes of different signalling pathways in this general model. The classification of inflammatory and mechanical damage could be coupled with mechanical stimulus in the future. Due to the existence of large uncertainty and variability on the parameters [43,44,76], the prediction of inflammation process might differ based on different parameters, and only the adipokine and mechanical damage parameters were tuned to study the effect of obesity and exercise. In turn, the stability analysis of predominant parameters can unravel the possibilities of the inflammation dynamics involved by obesity and physical activity. The prospective work can focus on the extension of this general model and specifying mediators so that there might be relevant data to validate its application of the prediction on inflammatory activities.

## Conclusion

A new mechanistic inflammation model of OA including the effect of obesity and exercise was developed to qualitatively analyse the dynamics of the chronic inflammatory process regulated by adipokines. Since tissue damage is the underlying trigger of inflammation, the production of MMPs was found to dominate the onset and development of inflammation comparing to pro-inflammatory cytokines under the regulation of adipokines. In addition, a BMI threshold of 33 was found to induce persistent inflammation. This threshold can vary depending on the individual parameter sets. The predominant role of adipokines in this model aggravates inflammatory damage but the reduction of obesity by physical activity intervention can regain the stability of healthy state. When the loss of system bistability results from mechanical damage, physical activity intervention can delay the activation of inflammation within a window period after the mechanical damage. This window period is determined by the speed of inflammation onset in general and it can provide insights on the timing of exercise therapy according to different obesity and damage levels. In the future, this model can be calibrated with specific molecular data and used to predict the inflammatory process regulated by obesity. Moreover, the mechanical response could be coupled to the current model through the production of fibronectin fragments to study the coeffects of inflammation and mechanics on OA onset and development.

## Supporting information

S1 Fig The comparison of bifurcation diagrams in the production of PICs between (a) Baker’s model and (b) the adipokine-mediated inflammation model. (TIFF)

S2 Fig The comparison of bifurcation diagrams in the production of AICs between (a) Baker’s model and (b) the adipokine-mediated inflammation model. (TIFF)

S3 Fig The comparison of bifurcation diagrams in the production of MMPs between (a) Baker’s model and (b) the adipokine-mediated inflammation model. (TIFF)

S4 Fig The comparison of bifurcation diagrams in the production of Fn-fs between (a) Baker’s model and (b) the adipokine-mediated inflammation model. (TIFF)

S5 Eqs The formula of parameter nondimensionalisation. (TIFF)

S6 Table The description of dimensional parameters. (TIFF)

S7 Eqs The simultaneous equations when production rates are at equilibrium for the calculation of fixed points. (TIFF)

S8 Fig The phase plane of the inflammation system under the baseline parameters: (a) Intersections of nullclines; (b) Trajectories in the phase plane. (TIFF)

## References

[1] King LK. Osteoarthritis and comorbidity: time for action. Osteoarthritis Cartilage. 2023;31(4):423–4. doi: 10.1016/j.joca.2023.01.007 36693559

[2] Cui A, Li H, Wang D, Zhong J, Chen Y, Lu H. Global, regional prevalence, incidence and risk factors of knee osteoarthritis in population-based studies. EClinicalMedicine. 2020;29–30:100587. doi: 10.1016/j.eclinm.2020.100587 34505846 PMC7704420

[3] James SL, Abate D, Abate KH, Abay SM, Abbafati C, Abbasi N, et al. Global, regional, and national incidence, prevalence, and years lived with disability for 354 diseases and injuries for 195 countries and territories, 1990-2017: a systematic analysis for the Global Burden of Disease Study 2017. Lancet. 2018;392(10159):1789–858. doi: 10.1016/S0140-6736(18)32279-7 30496104 PMC6227754

[4] Steinmetz JD, Culbreth GT, Haile LM, Rafferty Q, Lo J, Fukutaki KG, et al. Global, regional, and national burden of osteoarthritis, 1990-2020 and projections to 2050: a systematic analysis for the Global Burden of Disease Study 2021. Lancet Rheumatol. 2023;5(9):e508–22. doi: 10.1016/S2665-9913(23)00163-7 37675071 PMC10477960

[5] Shumnalieva R, Kotov G, Monov S. Obesity-Related Knee Osteoarthritis-Current Concepts. Life (Basel). 2023;13(8):1650. doi: 10.3390/life13081650 37629507 PMC10456094

[6] Park D, Park Y-M, Ko S-H, Hyun K-S, Choi Y-H, Min D-U, et al. Association of general and central obesity, and their changes with risk of knee osteoarthritis: a nationwide population-based cohort study. Sci Rep. 2023;13(1):3796. doi: 10.1038/s41598-023-30727-4 36882508 PMC9992488

[7] Urban H, Little CB. The role of fat and inflammation in the pathogenesis and management of osteoarthritis. Rheumatology (Oxford). 2018;57(suppl_4):iv10–21. doi: 10.1093/rheumatology/kex399 29444323

[8] González-Muniesa P, Mártinez-González M-A, Hu FB, Després J-P, Matsuzawa Y, Loos RJF, et al. Obesity. Nat Rev Dis Primers. 2017;3:17034. doi: 10.1038/nrdp.2017.34 28617414

[9] Andriacchi TP, Mündermann A, Smith RL, Alexander EJ, Dyrby CO, Koo S. A framework for the in vivo pathomechanics of osteoarthritis at the knee. Ann Biomed Eng. 2004;32(3):447–57. doi: 10.1023/b:abme.0000017541.82498.37 15095819

[10] MacLean KFE, Callaghan JP, Maly MR. Effect of obesity on knee joint biomechanics during gait in young adults. Cogent Med. 2016;3(1):1173778. doi: 10.1080/2331205x.2016.1173778

[11] Aaboe J, Bliddal H, Messier SP, Alkjær T, Henriksen M. Effects of an intensive weight loss program on knee joint loading in obese adults with knee osteoarthritis. Osteoarthritis Cartilage. 2011;19(7):822–8. doi: 10.1016/j.joca.2011.03.006 21440076

[12] Messier SP, Gutekunst DJ, Davis C, DeVita P. Weight loss reduces knee-joint loads in overweight and obese older adults with knee osteoarthritis. Arthritis Rheum. 2005;52(7):2026–32. doi: 10.1002/art.21139 15986358

[13] Glyn-Jones S, Palmer AJR, Agricola R, Price AJ, Vincent TL, Weinans H, et al. Osteoarthritis. Lancet. 2015;386(9991):376–87. doi: 10.1016/s0140-6736(14)60802-3 25748615

[14] Kolasinski SL, Neogi T, Hochberg MC, Oatis C, Guyatt G, Block J, et al. 2019 American College of Rheumatology/Arthritis Foundation Guideline for the Management of Osteoarthritis of the Hand, Hip, and Knee. Arthritis Care Res (Hoboken). 2020;72(2):149–62. doi: 10.1002/acr.24131 31908149 PMC11488261

[15] Bannuru RR, Osani MC, Vaysbrot EE, Arden NK, Bennell K, Bierma-Zeinstra SMA, et al. OARSI guidelines for the non-surgical management of knee, hip, and polyarticular osteoarthritis. Osteoarthritis Cartilage. 2019;27(11):1578–89. doi: 10.1016/j.joca.2019.06.011 31278997

[16] Chang J, Liao Z, Lu M, Meng T, Han W, Ding C. Systemic and local adipose tissue in knee osteoarthritis. Osteoarthritis Cartilage. 2018;26(7):864–71. doi: 10.1016/j.joca.2018.03.004 29578044

[17] Messier SP, Mihalko SL, Legault C, Miller GD, Nicklas BJ, DeVita P, et al. Effects of intensive diet and exercise on knee joint loads, inflammation, and clinical outcomes among overweight and obese adults with knee osteoarthritis: the IDEA randomized clinical trial. JAMA. 2013;310(12):1263–73. doi: 10.1001/jama.2013.277669 24065013 PMC4450354

[18] Masouros SD, Bull AMJ, Amis AA. (I) Biomechanics of the knee joint. Orthopaedics Trauma. 2010;24(2):84–91. doi: 10.1016/j.mporth.2010.03.005

[19] Duclos M. Osteoarthritis, obesity and type 2 diabetes: the weight of waist circumference. Ann Phys Rehabil Med. 2016;59(3):157–60. doi: 10.1016/j.rehab.2016.04.002 27211819

[20] Thompson D, Karpe F, Lafontan M, Frayn K. Physical activity and exercise in the regulation of human adipose tissue physiology. Physiol Rev. 2012;92(1):157–91. doi: 10.1152/physrev.00012.2011 22298655

[21] Rocha FAC, Ali SA. Soluble biomarkers in osteoarthritis in 2022: year in review. Osteoarthritis Cartilage. 2023;31(2):167–76. doi: 10.1016/j.joca.2022.09.005 36179981

[22] Papadopoulou SK, Papadimitriou K, Voulgaridou G, Georgaki E, Tsotidou E, Zantidou O, et al. Exercise and nutrition impact on osteoporosis and sarcopenia—the incidence of osteosarcopenia: a narrative review. Nutrients. 2021;13(12):4499. doi: 10.3390/nu13124499 34960050 PMC8705961

[23] Sanchez-Lopez E, Coras R, Torres A, Lane NE, Guma M. Synovial inflammation in osteoarthritis progression. Nat Rev Rheumatol. 2022;18(5):258–75. doi: 10.1038/s41584-022-00749-9 35165404 PMC9050956

[24] Sokolove J, Lepus CM. Role of inflammation in the pathogenesis of osteoarthritis: latest findings and interpretations. Ther Adv Musculoskelet Dis. 2013;5(2):77–94. doi: 10.1177/1759720X12467868 23641259 PMC3638313

[25] Kalaitzoglou E, Griffin TM, Humphrey MB. Innate immune responses and osteoarthritis. Curr Rheumatol Rep. 2017;19(8):45. doi: 10.1007/s11926-017-0672-6 28718060

[26] Kuettner KE, Cole AA. Cartilage degeneration in different human joints. Osteoarthritis Cartilage. 2005;13(2):93–103. doi: 10.1016/j.joca.2004.11.006 15694570

[27] Muldrew K. Osteoarthritis as an inevitable consequence of the structure of articular cartilage. Med Hypotheses. 2002;59(4):389–97. doi: 10.1016/s0306-9877(02)00122-6 12208177

[28] Lorenz H, Richter W. Osteoarthritis: cellular and molecular changes in degenerating cartilage. Prog Histochem Cytochem. 2006;40(3):135–63. doi: 10.1016/j.proghi.2006.02.003 16759941

[29] Sophia Fox AJ, Bedi A, Rodeo SA. The basic science of articular cartilage: structure, composition, and function. Sports Health. 2009;1(6):461–8. doi: 10.1177/1941738109350438 23015907 PMC3445147

[30] Wojdasiewicz P, Poniatowski ŁA, Szukiewicz D. The role of inflammatory and anti-inflammatory cytokines in the pathogenesis of osteoarthritis. Mediators Inflamm. 2014;2014:561459. doi: 10.1155/2014/561459 24876674 PMC4021678

[31] Burrage PS, Mix KS, Brinckerhoff CE. Matrix metalloproteinases: role in arthritis. Front Biosci. 2006;11:529–43. doi: 10.2741/1817 16146751

[32] Fingleton B. Matrix metalloproteinases as regulators of inflammatory processes. Biochim Biophys Acta Mol Cell Res. 2017;1864(11 Pt A):2036–42. doi: 10.1016/j.bbamcr.2017.05.010 28502592

[33] Mehana E-SE, Khafaga AF, El-Blehi SS. The role of matrix metalloproteinases in osteoarthritis pathogenesis: an updated review. Life Sci. 2019;234:116786. doi: 10.1016/j.lfs.2019.116786 31445934

[34] Unamuno X, Gómez-Ambrosi J, Rodríguez A, Becerril S, Frühbeck G, Catalán V. Adipokine dysregulation and adipose tissue inflammation in human obesity. Eur J Clin Invest. 2018;48(9):e12997. doi: 10.1111/eci.12997 29995306

[35] Poonpet T, Honsawek S. Adipokines: biomarkers for osteoarthritis? World J Orthop. 2014;5(3):319–27. doi: 10.5312/wjo.v5.i3.319 25035835 PMC4095025

[36] Francisco V, Pino J, Gonzalez-Gay MA, Mera A, Lago F, Gómez R, et al. Adipokines and inflammation: is it a question of weight? Br J Pharmacol. 2018;175(10):1569–79. doi: 10.1111/bph.14181 29486050 PMC5913397

[37] Zhang C, Lin Y, Yan CH, Zhang W. Adipokine signaling pathways in osteoarthritis. Front Bioeng Biotechnol. 2022;10:865370. doi: 10.3389/fbioe.2022.865370 35519618 PMC9062110

[38] Pérez-García S, Carrión M, Gutiérrez-Cañas I, Villanueva-Romero R, Castro D, Martínez C, et al. Profile of matrix-remodeling proteinases in osteoarthritis: impact of fibronectin. Cells. 2019;9(1):40. doi: 10.3390/cells9010040 31877874 PMC7017325

[39] Collins KH, Lenz KL, Pollitt EN, Ferguson D, Hutson I, Springer LE, et al. Adipose tissue is a critical regulator of osteoarthritis. Proc Natl Acad Sci U S A. 2021;118(1):e2021096118. doi: 10.1073/pnas.2021096118 33443201 PMC7817130

[40] Dumond H, Presle N, Terlain B, Mainard D, Loeuille D, Netter P, et al. Evidence for a key role of leptin in osteoarthritis. Arthritis Rheum. 2003;48(11):3118–29. doi: 10.1002/art.11303 14613274

[41] Hülser M-L, Luo Y, Frommer K, Hasseli R, Köhler K, Diller M, et al. Systemic versus local adipokine expression differs in a combined obesity and osteoarthritis mouse model. Sci Rep. 2021;11(1):17001. doi: 10.1038/s41598-021-96545-8 34417537 PMC8379250

[42] Kroon FPB, Veenbrink AI, de Mutsert R, Visser AW, van Dijk KW, le Cessie S, et al. The role of leptin and adiponectin as mediators in the relationship between adiposity and hand and knee osteoarthritis. Osteoarthritis Cartilage. 2019;27(12):1761–7. doi: 10.1016/j.joca.2019.08.003 31450004

[43] Baker M, Brook BS, Owen MR. Mathematical modelling of cytokines, MMPs and fibronectin fragments in osteoarthritic cartilage. J Math Biol. 2017;75(4):985–1024. doi: 10.1007/s00285-017-1104-y 28213682 PMC5562782

[44] Rahman MM, Watton PN, Neu CP, Pierce DM. A chemo-mechano-biological modeling framework for cartilage evolving in health, disease, injury, and treatment. Comput Methods Programs Biomed. 2023;231:107419. doi: 10.1016/j.cmpb.2023.107419 36842346

[45] Kar S, Smith DW, Gardiner BS, Li Y, Wang Y, Grodzinsky AJ. Modeling IL-1 induced degradation of articular cartilage. Arch Biochem Biophys. 2016;594:37–53. doi: 10.1016/j.abb.2016.02.008 26874194

[46] Graham JM, Ayati BP, Ding L, Ramakrishnan PS, Martin JA. Reaction-diffusion-delay model for EPO/TNF-α interaction in articular cartilage lesion abatement. Biol Direct. 2012;7:9. doi: 10.1186/1745-6150-7-9 22353555 PMC3356234

[47] Campbell K, Naire S, Kuiper JH. A mathematical model of cartilage regeneration after chondrocyte and stem cell implantation - I: the effects of growth factors. J Tissue Eng. 2019;10:2041731419827791. doi: 10.1177/2041731419827791 30906518 PMC6421619

[48] Wang X, Brouillette MJ, Ayati BP, Martin JA. A validated model of the pro- and anti-inflammatory cytokine balancing act in articular cartilage lesion formation. Front Bioeng Biotechnol. 2015;3:25. doi: 10.3389/fbioe.2015.00025 25806365 PMC4354422

[49] Kapitanov GI, Wang X, Ayati BP, Brouillette MJ, Martin JA. Linking cellular and mechanical processes in articular cartilage lesion formation: a mathematical model. Front Bioeng Biotechnol. 2016;4:80. doi: 10.3389/fbioe.2016.00080 27843894 PMC5086581

[50] Wang X, Ayati BP, Brouillete MJ, Graham JM, Ramakrishnan PS, Martin JA. Modeling and simulation of the effects of cyclic loading on articular cartilage lesion formation. Int J Numer Method Biomed Eng. 2014;30(10):927–41. doi: 10.1002/cnm.2636 24753483 PMC4950512

[51] Pollatschek MA, Nahir AM. A mathematical model of osteoarthosis. J Theor Biol. 1990;143(4):497–505. doi: 10.1016/s0022-5193(05)80026-8 2381242

[52] Weber M-C, Fischer L, Damerau A, Ponomarev I, Pfeiffenberger M, Gaber T, et al. Macroscale mesenchymal condensation to study cytokine-driven cellular and matrix-related changes during cartilage degradation. Biofabrication. 2020;12(4):045016. doi: 10.1088/1758-5090/aba08f 32598334

[53] Lesage R, Ferrao Blanco MN, Narcisi R, Welting T, van Osch GJVM, Geris L. An integrated in silico-in vitro approach for identifying therapeutic targets against osteoarthritis. BMC Biol. 2022;20(1):253. doi: 10.1186/s12915-022-01451-8 36352408 PMC9648005

[54] Segarra-Queralt M, Neidlin M, Tio L, Monfort J, Monllau JC, González Ballester MÁ, et al. Regulatory network-based model to simulate the biochemical regulation of chondrocytes in healthy and osteoarthritic environments. Sci Rep. 2022;12(1):3856. doi: 10.1038/s41598-022-07776-2 35264634 PMC8907219

[55] Ferrao Blanco MN, Lesage R, Kops N, Fahy N, Bekedam FT, Chavli A, et al. A multi-model approach identifies ALW-II-41-27 as a promising therapy for osteoarthritis-associated inflammation and endochondral ossification. Heliyon. 2024;10(23):e40871. doi: 10.1016/j.heliyon.2024.e40871 39717596 PMC11664402

[56] Segarra-Queralt M, Crump K, Pascuet-Fontanet A, Gantenbein B, Noailly J. The interplay between biochemical mediators and mechanotransduction in chondrocytes: unravelling the differential responses in primary knee osteoarthritis. Phys Life Rev. 2024;48:205–21. doi: 10.1016/j.plrev.2024.02.003 38377727

[57] Segarra-Queralt M, Piella G, Noailly J. Network-based modelling of mechano-inflammatory chondrocyte regulation in early osteoarthritis. Front Bioeng Biotechnol. 2023;11:1006066. doi: 10.3389/fbioe.2023.1006066 36815875 PMC9936426

[58] Baker M, Denman-Johnson S, Brook BS, Gaywood I, Owen MR. Mathematical modelling of cytokine-mediated inflammation in rheumatoid arthritis. Math Med Biol. 2013;30(4):311–37. doi: 10.1093/imammb/dqs026 23002057

[59] Robinson WH, Lepus CM, Wang Q, Raghu H, Mao R, Lindstrom TM, et al. Low-grade inflammation as a key mediator of the pathogenesis of osteoarthritis. Nat Rev Rheumatol. 2016;12(10):580–92. doi: 10.1038/nrrheum.2016.136 27539668 PMC5500215

[60] Liu C, Chu D, Kalantar-Zadeh K, George J, Young HA, Liu G. Cytokines: from clinical significance to quantification. Adv Sci (Weinh). 2021;8(15):e2004433. doi: 10.1002/advs.202004433 34114369 PMC8336501

[61] Wang T, He C. Pro-inflammatory cytokines: the link between obesity and osteoarthritis. Cytokine Growth Factor Rev. 2018;44:38–50. doi: 10.1016/j.cytogfr.2018.10.002 30340925

[62] Santillán M. On the use of the hill functions in mathematical models of gene regulatory networks. Math Model Nat Phenom. 2008;3(2):85–97. doi: 10.1051/mmnp:2008056

[63] Spalding KL, Arner E, Westermark PO, Bernard S, Buchholz BA, Bergmann O, et al. Dynamics of fat cell turnover in humans. Nature. 2008;453(7196):783–7. doi: 10.1038/nature06902 18454136

[64] Palacios-Marin I, Serra D, Jimenez-Chillarón J, Herrero L, Todorčević M. Adipose tissue dynamics: cellular and lipid turnover in health and disease. Nutrients. 2023;15(18):3968. doi: 10.3390/nu15183968 37764752 PMC10535304

[65] Brooks GA, Butte NF, Rand WM, Flatt J-P, Caballero B. Chronicle of the institute of medicine physical activity recommendation: how a physical activity recommendation came to be among dietary recommendations. Am J Clin Nutr. 2004;79(5):921S-930S. doi: 10.1093/ajcn/79.5.921S 15113740

[66] van Riel NAW. Dynamic modelling and analysis of biochemical networks: mechanism-based models and model-based experiments. Brief Bioinform. 2006;7(4):364–74. doi: 10.1093/bib/bbl040 17107967

[67] Chen T-H, Chen L, Hsieh M-S, Chang C-P, Chou D-T, Tsai S-H. Evidence for a protective role for adiponectin in osteoarthritis. Biochim Biophys Acta. 2006;1762(8):711–8. doi: 10.1016/j.bbadis.2006.06.008 16891099

[68] Hawker GA, Stewart L, French MR, Cibere J, Jordan JM, March L, et al. Understanding the pain experience in hip and knee osteoarthritis--an OARSI/OMERACT initiative. Osteoarthritis Cartilage. 2008;16(4):415–22. doi: 10.1016/j.joca.2007.12.017 18296075

[69] Mahmoudian A, Lohmander LS, Mobasheri A, Englund M, Luyten FP. Early-stage symptomatic osteoarthritis of the knee-time for action. Nat Rev Rheumatol. 2021;17(10):621–32. doi: 10.1038/s41584-021-00673-4 34465902

[70] Iolascon G, Gimigliano F, Moretti A, de Sire A, Migliore A, Brandi ML, et al. Early osteoarthritis: How to define, diagnose, and manage. A systematic review. Eur Geriatric Med. 2017;8(5–6):383–96. doi: 10.1016/j.eurger.2017.07.008

[71] Herrero-Manley L, Alabajos-Cea A, Suso-Martí L, Viosca-Herrero E. Classification criteria for early knee osteoarthritis: a review article. Aktuel Rheumatol. 2023;49(06):365–75. doi: 10.1055/a-2173-1607

[72] Sandell LJ, Aigner T. Articular cartilage and changes in arthritis. An introduction: cell biology of osteoarthritis. Arthritis Res. 2001;3(2):107–13. doi: 10.1186/ar148 11178118 PMC128887

[73] Felson DT. Osteoarthritis as a disease of mechanics. Osteoarthritis Cartilage. 2013;21(1):10–5. doi: 10.1016/j.joca.2012.09.012 23041436 PMC3538894

[74] Van Spil WE, Kubassova O, Boesen M, Bay-Jensen A-C, Mobasheri A. Osteoarthritis phenotypes and novel therapeutic targets. Biochem Pharmacol. 2019;165:41–8. doi: 10.1016/j.bcp.2019.02.037 30831073

[75] Berenbaum F, Sellam J. Obesity and osteoarthritis: what are the links? Joint Bone Spine. 2008;75(6):667–8. doi: 10.1016/j.jbspin.2008.07.006 18990601

[76] Moise N, Friedman A. Rheumatoid arthritis—a mathematical model. J Theor Biol. 2019;461:17–33. doi: 10.1016/j.jtbi.2018.10.039 30347191

