# Supplementary figures and images for: Mathematical modelling of inflammatory process and obesity in osteoarthritis
Source: PLoS One. 2025 Jun 2;20(6):e0323258. doi: 10.1371/journal.pone.0323258 (PMC12129326; doi:10.1371/journal.pone.0323258)

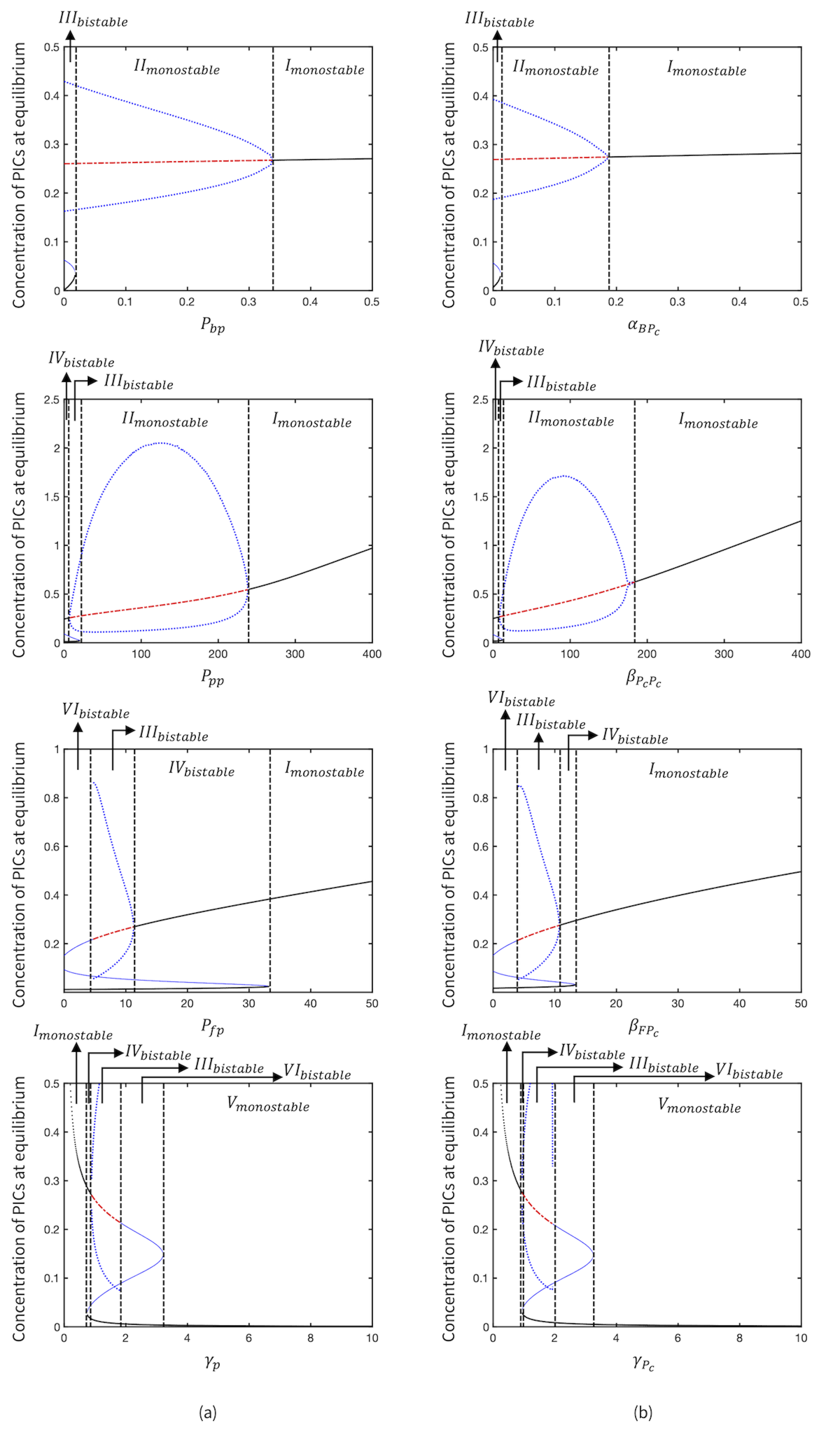

Supplement: S1 Fig — (TIFF) [file pone.0323258.s001.tif]

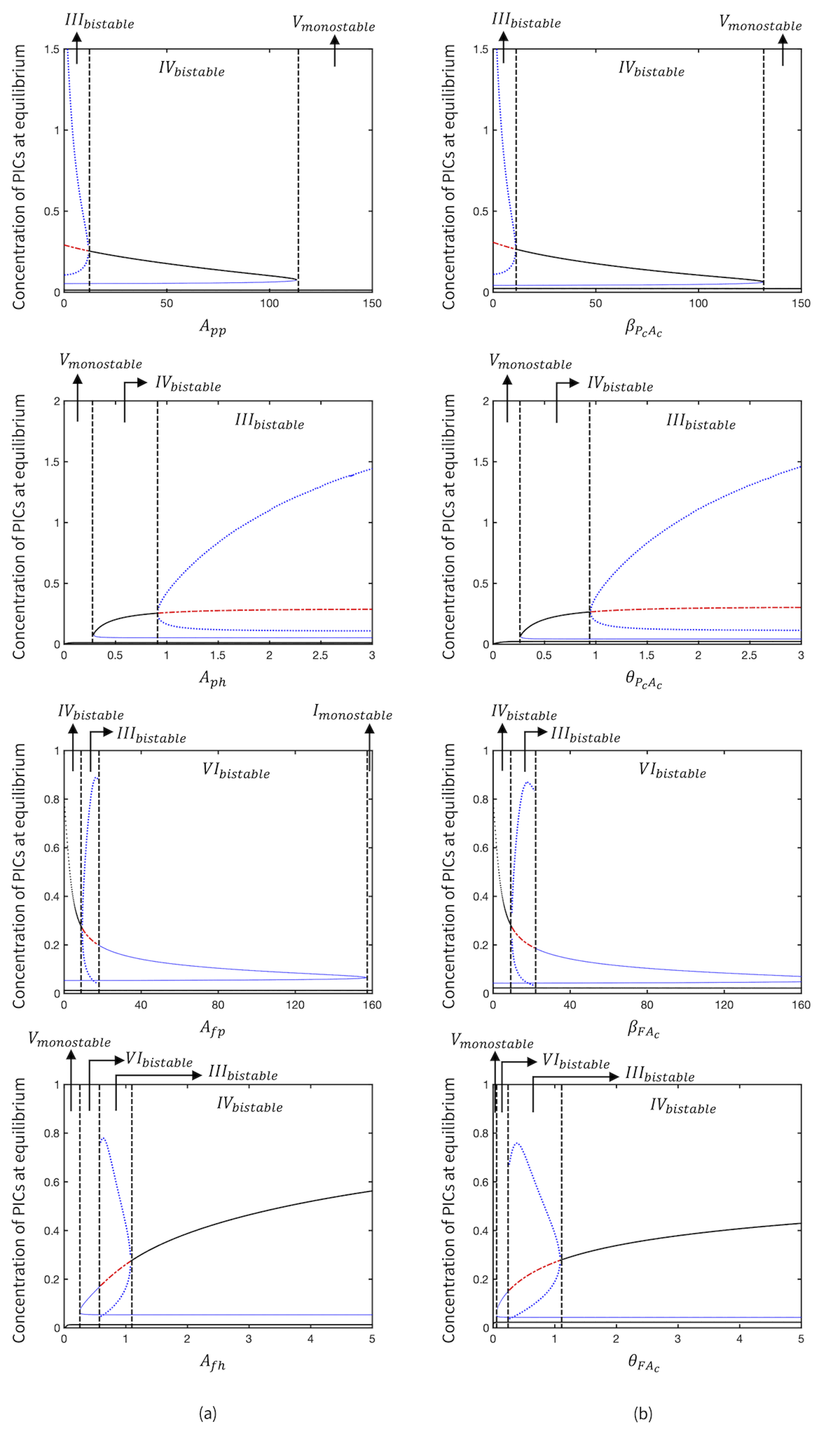

Supplement: S2 Fig — (TIFF) [file pone.0323258.s002.tif]

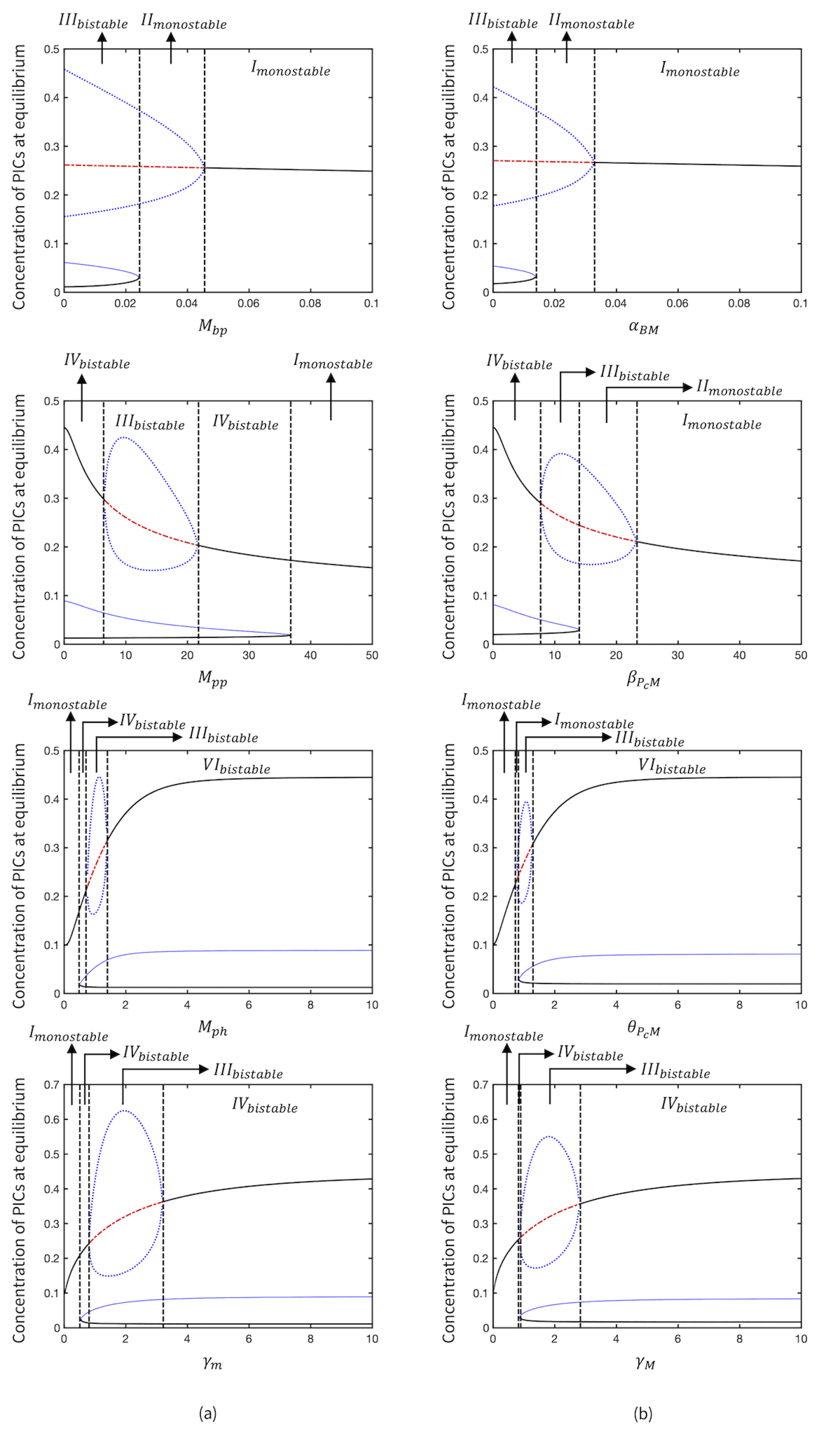

Supplement: S3 Fig — (TIFF) [file pone.0323258.s003.tif]

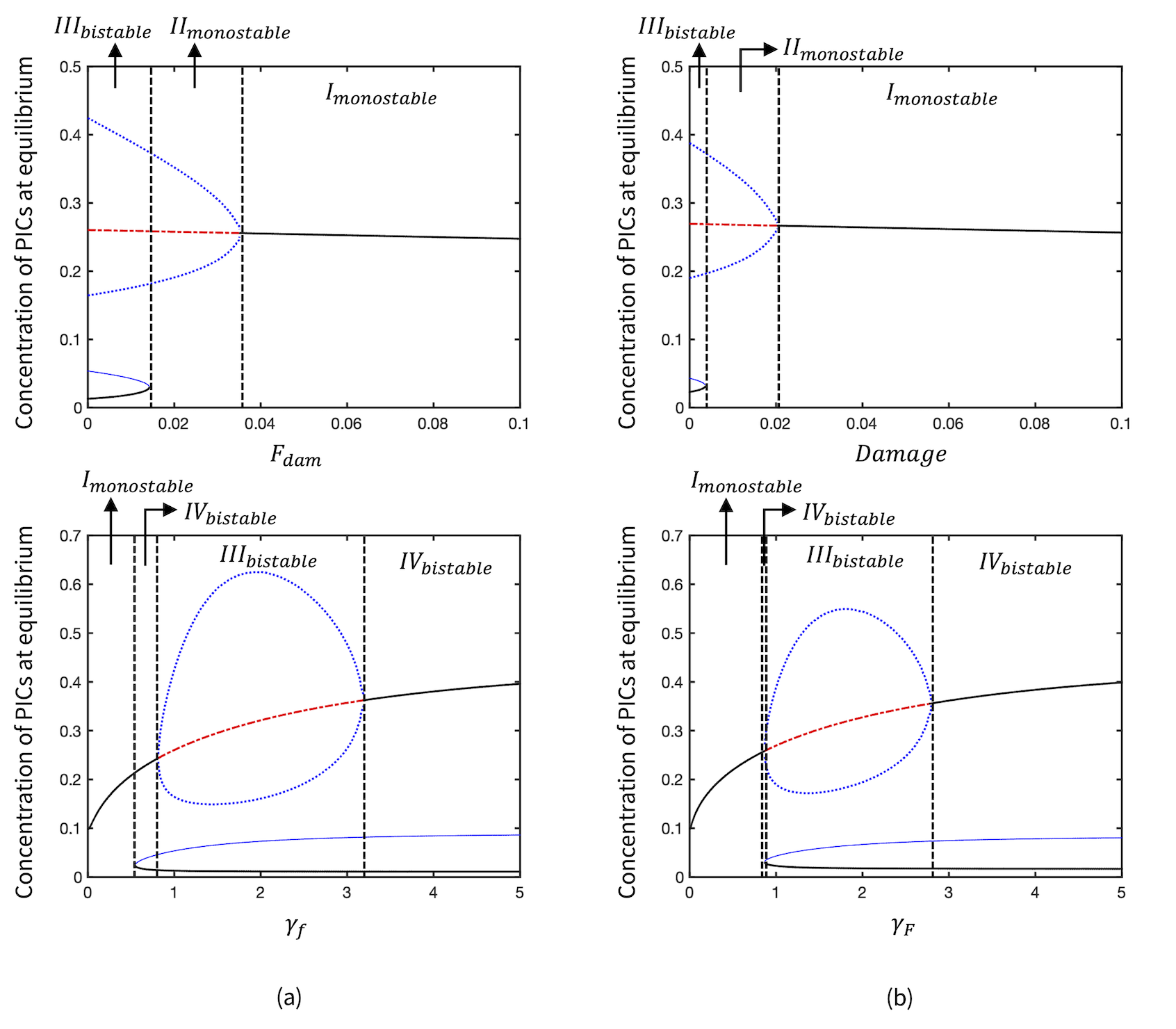

Supplement: S4 Fig — (TIFF) [file pone.0323258.s004.tif]

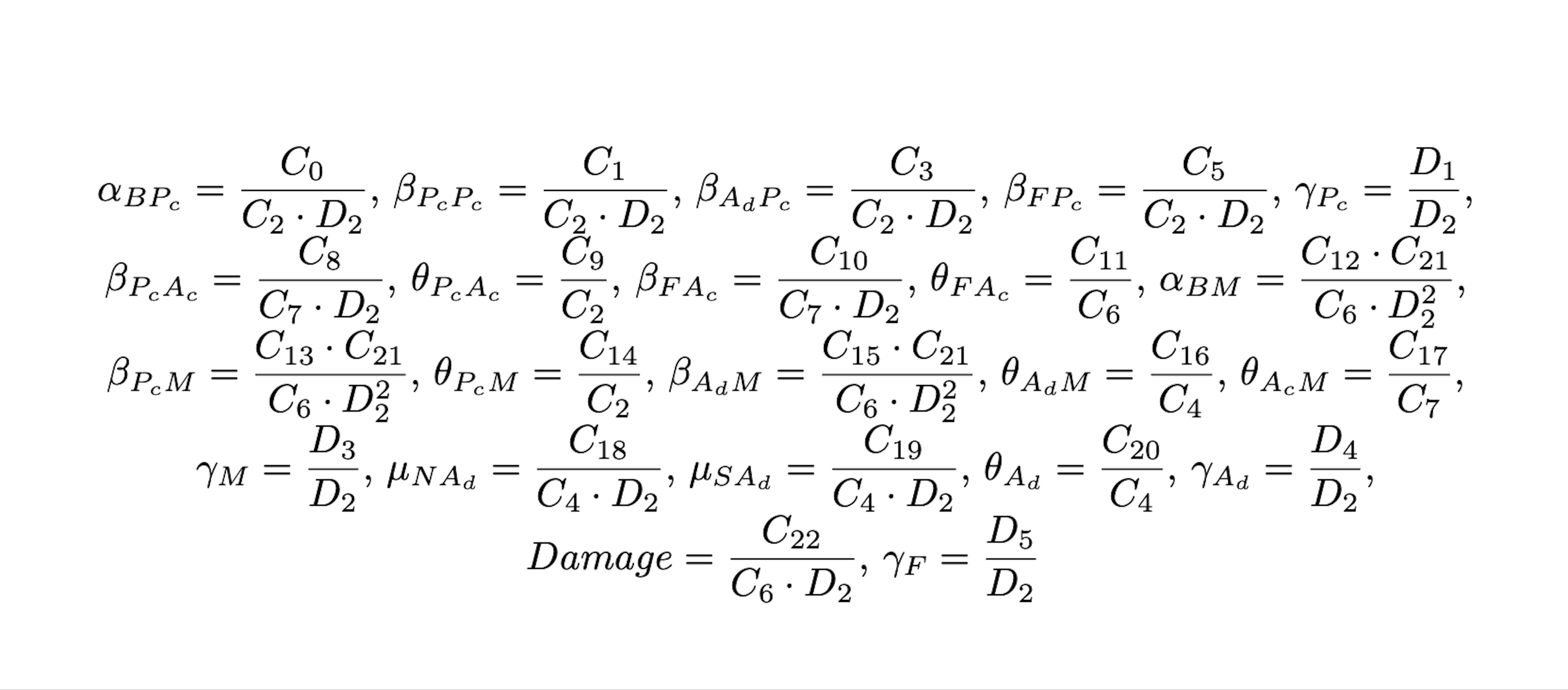

Supplement: S5 Eqs — (TIFF) [file pone.0323258.s005.tif]

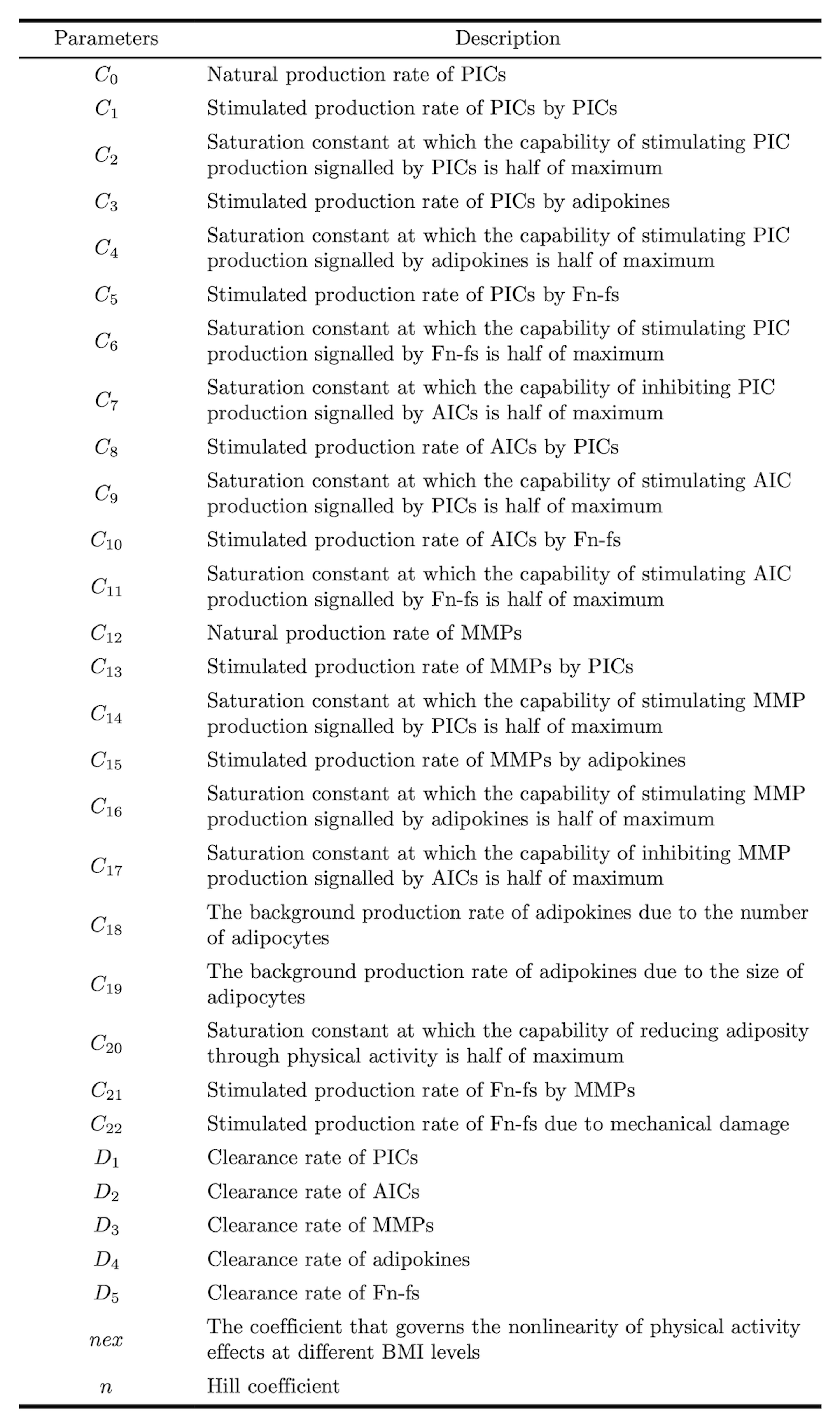

Supplement: S6 Table — (TIFF) [file pone.0323258.s006.tif]

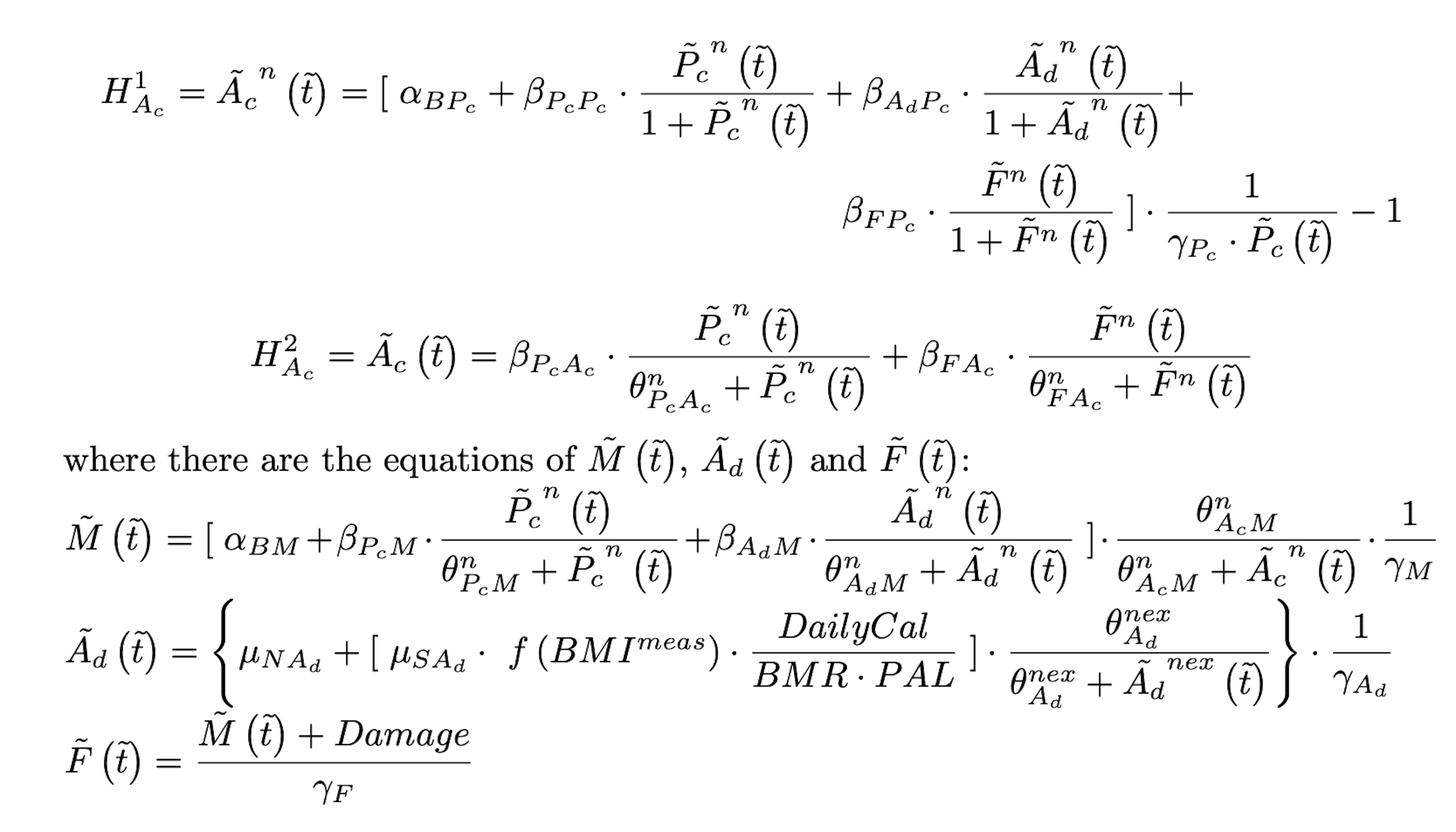

Supplement: S7 Eqs — (TIFF) [file pone.0323258.s007.tif]

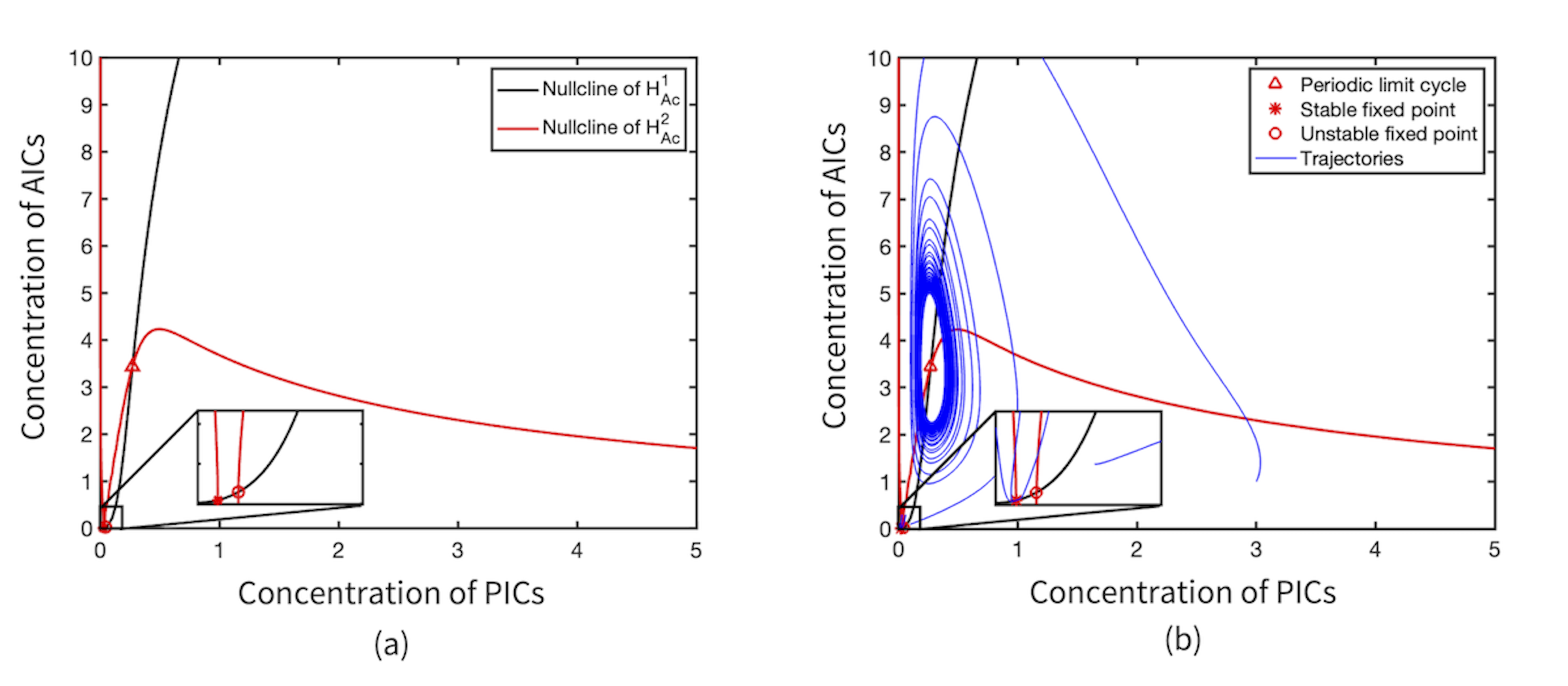

Supplement: S8 Fig — (TIFF) [file pone.0323258.s008.tif]
